# Ouabain – a double-edged sword in tumor development and progression? a review of half a century

**DOI:** 10.3389/fphys.2025.1685871

**Published:** 2025-10-03

**Authors:** Heidrun Weidemann, Alaa Daoud Sarsour, Chaya Brodie

**Affiliations:** ^1^ Department Internal Medicine, St. Georg Hospital, Eisenach, Germany; ^2^ The Mina and Everard Goodman Faculty of Life Sciences, Bar-Ilan University, Ramat Gan, Israel; ^3^ Institute of Nanotechnology and Advanced Materials (BINA), Bar-Ilan University, Ramat Gan, Israel

**Keywords:** ouabain, Na K ATPase, tumorigenesis, tumor escape, senolysis, apoptosis, anoikosis

## Abstract

Since their first discovery as potential anti-cancer drugs there is increasing evidence that cardiotonic steroids e.g., Ouabain have anti-tumor properties by interacting with their natural receptor the Na^+^-K^+^-ATPase (NKA) and by inducing diverse intracellular signaling pathways. It is well established that the NKA represents a signal transducer that is partly independent from its pump activity. In the early 90ies endogenous Ouabain (EO) was discovered in the serum of different species, including human beings. It was demonstrated that Ouabain is synthesized and released from the adrenal gland. The concept of endogenous Ouabain as a “stress hormone” playing important roles in the regulation of hypertension, volume homeostasis, cardiac function and, last but not least, cancer was established. We developed the hypothesis that long-lasting stress with adrenal exhaustion i.e., very low endogenous Ouabain levels may predispose to tumorigenesis. On the contrary, some authors recently have questioned the tumor-protective role of Ouabain and claimed that endogenous Ouabain promotes tumor escape mechanisms. In order to clarify these and other opposing or contradictious data we will summarize in this review PubMed data from the last 50 years about “Ouabain and cancer”. We will demonstrate that overwhelming evidence speaks in favor of an anti-tumor effect of Ouabain. Exogenous Ouabain has been shown to be identical to endogenous Ouabain, hence we conclude that a potential harmful role of endogenous Ouabain is minor compared to the huge potential benefit of Ouabain in defeating and suppressing the development of cancer.

## 1 Introduction

Since their first discovery as potential anti-cancer drugs 3 decades ago, Repke et al. (1995), Haux (1999), Newman et al. (2008) there is increasing evidence over the last years, in-vitro and in-vivo, that cardiotonic steroids (CTS) have anti-tumor properties by interacting with their natural receptor the Na^+^-K^+^-ATPase (NKA) not only via the classical way i.e., NKA inhibition but via inducing diverse intracellular signaling pathways (Johansson et al., 2001; Winnicka et al., 2006; López-Lázaro, 2007). It is meanwhile established that the NKA represents a signal transducer that is partly independent from its pump activity (Xie et al., 2003; Aizman and Aperia, 2003; Liang et al., 2007). In this review we will focus on Ouabain because it is so far the best studied CTS *in vitro* as well as *in vivo* studies dealing with their potential as anti-cancer compounds. Ouabain stems from an African tree (Acokanthera ouabaio) and is used as arrow poison Five decades ago exogenous Ouabain was often used to study the altered ion transport mechanisms in tumor cells, the most established models were Ehrlich ascites cells (Mayhew, 1972) and chicken embryo cells transformed by the Rous sarcoma virus (Banerjee et al., 1977). Mainly, an increased Ouabain-sensitive K^+^-influx measured by 86Rb+ uptake was observed but also a higher intracellular Na^+^ level due to increased permeability of the plasma cell membrane supposedly induced by a virus-coded transformation-inducing protein (Kimelberg and Mayhew, 1975; Shen et al., 1978; Johnson and Weber, 1979; Ba et al., 1981). This leakage theory is crucial for the altered higher activity of the Na^+^-/K^+^-ATPase in tumor cells, a key feature of transforming cells to provide the necessary energy e.g., for new protein synthesis (Kimelberg and Mayhew, 1976). Interestingly, also the glucose up-take is increased in e.g., Rous sarcoma virus transformed cells (Bader et al., 1981; Weber et al., 1984). In this context the role of (high) NKA activity in aerobic glycolysis (Warburg effect) - a hallmark of cancer metabolism - was discovered (Racker, 1976; Racker et al., 1983; Balaban and Bader, 1984; Vaupel et al., 2019). Interestingly, the high NKA activity doesn’t seem to stem from a less efficient pump in transformed cells (Balaban and Bader, 1983). In summary, these studies hinted to a suppressive effect of Ouabain/EO on tumorigenesis at an early stage. In the 80ies/early 90ies endogenous Ouabain (EO) was discovered in the serum of different species, including human beings (Hamlyn et al., 1991). It was demonstrated that Ouabain is synthesized and released from the adrenal gland (Doris, 1988; Laredo et al., 1994) rejecting the claim of critics that authentic Ouabain might be stored there after digestion (Doris et al., 1996). However, until today a dispute is ongoing whether endogenous Ouabain exists at all resp. whether EO is identical to Ouabain (Lewis et al., 2014; Kaaja and Nicholls, 2018). One study of special importance we will mention in detail. Baecher et al. (2014) claimed that the immunoassays so far used were not standardized. They developed an ultra-sensitive and highly specific method for measurement of Ouabain in human plasma based on isotope dilution liquid chromatography tandem-mass spectrometry (UP/ID-LC-MS/MS). After validation of the method which had a lower quantification limit of 1,7 pmol/L Ouabain was not observed in plasma samples from patients with and without heart failure. The authors concluded that immunoassays previously used to quantify assumed EO detected compounds which are not structurally identical with Ouabain (Baecher et al., 2014). Ferrandi et al. (1997) saw different results, maybe due to a less specific method: they identified by single high-performance liquid chromatography (HPLC) a fraction identical to that of Ouabain and quantified it by two assay methods (radioimmunoassay and ATPase assay) in human plasma. Interestingly, recovery of standard Ouabain in pre-HPLC plasma extracts was approximately 90% and pre-HPLC Ouabain concentrations in human plasma ranged between 0.05 and 0.75 nmol/L (=50–750 pmol/L) (Ferrandi et al., 1997). We agree that cross-reactivity of structurally similar Ouabain-like compounds of endogenous origin may cause these discrepancies between immunoassays and mass spectrometric analyses. For this reason many authors prefer nowadays to speak of endogenous Ouabain-like compounds (OLC). We use here the terms OLC and EO as synonyms.

The concept of endogenous Ouabain as a multifaceted hormone playing important roles in the regulation of hypertension, volume homeostasis, cardiac function and, last but not least, cancer was established (Blaustein, 1996; Manunta et al., 2009; Chen et al., 2006). We developed the hypothesis that long-lasting stress with adrenal exhaustion i.e., very low endogenous Ouabain levels may predispose to tumorigenesis (Weidemann, 2005). This is a crucial point because recently authors have questioned the tumor-protective role of endogenous Ouabain and claimed that, on the contrary, the endogenous ligand of NKA α1, EO, promotes tumor escape mechanisms (Yang et al., 2023). Whereas, indeed, there are abundant *in vitro* and (less) *in vivo* data about the (tumor-suppressive) effects of exogenous Ouabain on diverse cancer cell lines there are only few data about endogenous Ouabain, their plasma levels in humans and especially in cancer patients as well as their actions (Komiyama et al., 1999; Balzan et al., 2001; Schoner, 2002; Manunta et al., 2005; Pitzalis et al., 2006; Stella et al., 2008; El-Mallakh et al., 2010; Bignami et al., 2013). One reason for this relative lack of data might be that until today there is no commercial kit available to measure (routinely) endogenous Ouabain levels (see above). Here a new valid method is urgently needed to measure and track endogenous Ouabain plasma levels and exogenous applied Ouabain. Currently we are planning to develop an aptamer for Ouabain resp. EO to analyze their signaling pathways and cell metabolism. Aptamers are ssDNA or RNA oligonucleotides that bind with high affinity and specificity to a target molecule. Hence, aptamers are considered as alternatives to antibodies and are very useful for biosensor applications (Song et al., 2012) or as therapeutic agents (Nimjee et al., 2017; Menger et al., 2006). Aptamers are isolated by an *in vitro* process called “systematic evolution of ligands by exponential enrichment” (SELEX) (Ellington and Szostak, 1990). In this complex process an aptamer library is screened for sequences that have an affinity for a given target molecule. Numerous analytical techniques, such as electrochemical, colorimetric, optical methods can be applied to detect targets, due to convenient modifications and the stability of aptamers (Dreymann et al., 2022). With this new tool it would be possible to explore simultaneously the dose- and time dependant interactions of EO and exogenous Ouabain (resulting e.g., in disaggregation of NKA tetraprotomers). According to Blaustein and Hamlyn (2024) the understanding of this interaction is crucial for the potential use of Ouabain and other CTS in cancer therapy (Blaustein and Hamlyn, 2024).

## 2 Aim

1. We will list the anti-tumor signaling pathways of Ouabain (by giving one example) with describing more in detail some novel only recently revealed mechanisms.
2. We will summarize the sometimes indeed contradicting data about the effects of (exogenous) Ouabain on cancer including hematological malignant cell lines. It will be shown that with overwhelming evidence the anti-cancer effects of Ouabain are confirmed (mainly by in-vitro but also in-vivo data).
3. We will evaluate the data about new emerging effects of (endogenous) Ouabain on the immune system. It will be shown that the majority of data speak in favor of a tumor-suppressive effect of Ouabain.
4. We will describe the data dealing with the expression of NKA isoforms in tumor tissues and the role they are supposed to play in tumorigenesis. It will be shown that in tumor tissue often a switch from NKAα1 to NKAα3 expression occurs eventually induced by EO.
5. Connected to this issue (NKA expression) we will outline the data dealing with obvious different effects of Ouabain on malignant as compared to benign cells.
6. We will outline and discuss the dual role of the Scr-ERK1/2 activation by Ouabain in cancer. We will show that ERK1/2 activation indeed can induce proliferation in malignant cells but that especially a sustained ERK1/2 activation results in cell growth arrest.
7. We will cite a few studies analyzing the metabolic influences of Ouabain on cancer. It will be revealed that Ouabain inhibits the high glycolysis rate in cancer cells.
8. We shortly will summarize data about the effects of Ouabain on drug resistance mechanisms. Here it will be shown that there might be indeed a negative impact leading to increased drug resistance.

## 3 Results

### 3.1 Anti-tumor mechanisms of CTS with focus on ouabain

#### 3.1.1 Induction of apoptosis by (the following listed mechanisms)

##### 3.1.1.1 Modulation of intracellular Ca^++^ levels

McConkey et al. (2000) reported that the cardiac glycosides Oleandrin, Ouabain, and Digoxin induce apoptosis in androgen-independent human prostate cancer cell lines *in vitro*. Single-cell imaging of intracellular Ca^++^ fluxes demonstrated that the pro-apoptotic effects of the cardiac glycosides were linked to their abilities to induce sustained Ca^++^ increases in the cells. (Yeh et al., 2001; Huang et al., 2004; Winnicka et al., 2007; Xu et al., 2010; Chou et al., 2018; Guo et al., 2019; McConkey et al., 2000; Yeh et al., 2001; Huang et al., 2004; Winnicka et al., 2007; Xu et al., 2010; Chou et al., 2018; Guo et al., 2019; McConkey et al., 2000; Yeh et al., 2001; Huang et al., 2004; Winnicka et al., 2007; Xu et al., 2010; Chou et al., 2018; Guo et al., 2019; McConkey et al., 2000).

##### 3.1.1.2 Increasing intracellular ROS

Reactive oxygen species (ROS) comprise e.g., superoxide anion (O2^-^), hydrogen peroxide (H_2_O_2_) and hydroxyl radical (^.^HO). While at low physiologic levels they support the capability of the cells to adapt to intracellular stress an increased ROS level leads to molecular damage, ‘oxidative distress,’ an effect used for targeting tumor cells. Yan et al. (2015) investigated the molecular mechanism involved in Ouabain-induced ROS generation and cell apoptosis on human U373MG and U87MG glioma cells (Yan et al., 2015). Reducing ROS by three different ROS scavenger partly reversed Ouabains effect on cell apoptosis. Ouabain-induced ROS generation was regulated by p66Shc Ser36 phosphorylation. Knockdown of p66Shc by siRNA significantly inhibited ROS generations induced by Ouabain (Huang et al., 2004; Kim et al., 2016; Chang et al., 2019).

##### 3.1.1.3 TRAIL

The tumor necrosis factor-related apoptosis-inducing ligand (TRAIL) is a crucial protein mainly at the cell surface of natural killer (NK) cells and macrophages which, after binding to its receptors e.g., on tumor cells, induces programmed cell death via forming the death-inducing signaling complex (DISC). Chanvorachote and Pongrakhananon (2013) stressed that resistance to TRAIL is a prerequisite for cancer progression, and TRAIL resistance is prevalent especially in lung cancer. Nontoxic concentrations of Ouabain were shown to increase caspase-3 activation, poly (ADP-ribose) polymerase (PARP) cleavage and apoptosis of H292 lung cancer cells in response to TRAIL (Chanvorachote and Pongrakhananon, 2013). Remarkably, Ouabain had a minimal effect on Bcl-2 and Bax levels, but induces the downregulation of the anti-apoptotic Mcl-1 protein. The authors showed that the TRAIL-sensitizing effect of Ouabain is associated with its ability to generate reactive oxygen species (ROS), which trigger the proteasomal Mcl-1 degradation.

##### 3.1.1.4 Bcl-2 and Mcl-1 downregulation

The family of Bcl-2 proteins plays an important role in modulating apoptosis and are comprised of 3 groups, the anti-apoptotic Bcl-2, Mcl-1 and Bcl-xL, the pro-apoptotic executive Bax, Bak and Bok, and the pro-apoptotic sensor proteins Bim, Bid, Bad, Puma and Noxa. Meng et al. (2016) demonstrated that Ouabain treatment of Burkitt lymphoma Raji cells significantly inhibited cell proliferation in a dose-dependent manner and increased both early and late apoptosis. Increased levels of caspase-3 and cleaved-caspase-3, higher Bax activity and decreased expression of Bcl-2 were detected in Ouabain-treated Raji cells (Meng et al., 2016). Moreover, the observation of vacuole accumulation in Ouabain-treated Raji cells indicated that these cells were undergoing autophagy, correlating with the upregulation of the autophagy-related proteins LC3-II and Beclin-1 (Chanvorachote and Pongrakhananon, 2013; Cerella et al., 2015; Trenti et al., 2014; Wang et al., 2021).

##### 3.1.1.5 STAT-3 downregulation

Signal transducer and activator of transcription 3 (STAT3) is a cytoplasmic transcriptional factor involved in almost all cancer activities including tumor proliferation, metastasis, angiogenesis, immunosuppression, tumor inflammation, metabolism reprogramming, drug resistance, and cancer stemness. Du et al. (2021) revealed in different human cancer cell lines (non-small-cell lung cancer A549 and H460, colorectal carcinoma HCT116, pancreatic cacinoma PANC1 and cervical cancer Hela) an increase in apoptosis, intracellular ROS generation and DNA double-strand breaks induced by Ouabain treatment (Du et al., 2021). Besides, Ouabain effectively suppressed STAT3 expression as well as phosphorylation in addition to blocking STAT3-mediated transcription and downstream target proteins. Interestingly, these inhibitory activities seemed to be independent of the Na^+^/K^+^-ATPase.

#### 3.1.2 Induction of autophagy

Autophagy is a caspase-3 independent cell death. Wang et al. (2012) investigated the anti-cancer effects of Digoxin and Ouabain in A549 and H460 lung cancer cell lines. Remarkably, both CTS caused significant growth inhibition at nanomolar concentrations and moderate G2/M arrest but not apoptosis at IC50 levels (Wang et al., 2012). Moreover, autophagy was markedly induced by both compounds as proven by increase of autophagy markers. Importantly, the AMP-activated protein kinase (AMP-K) pathway was activated, resulting in mammalian target of rapamycin (mTOR) deactivation during autophagy induction. Moreover, extracellular-signal-regulated kinase 1/2 (ERK1/2) activation was found to be involved in the autophagy regulation. Co-treatment with inhibitors or siRNAs blocked the autophagic phenotypes and significantly increased cellular viability (Trenti et al., 2014; Meng et al., 2016; Wang et al., 2015; Škubník et al., 2021a).

#### 3.1.3 Induction of hybrid cell death

Hybrid cell death is defined as a form of concurrent apoptosis and necrosis. Chen et al. (2014) measured cell death and signaling pathways as well as intracellular Ca^++^, Na^+^ and K^+^ changes in two human glioblastoma (GBM) cell lines, LN229 and Temozolomide (TMZ)-resistant T98G after treatment with Ouabain (0,1–10 μM) (Chen et al., 2014). Remarkable was a disruption of K^+^ homeostasis. An apoptotic component was accompanied by reduced Bcl-2 expression and mitochondrial membrane potential. Electron microscopy revealed both apoptotic and necrotic alterations in the same cells. The authors found a high expression of the NKA α3 isoform in human T98G glioblastoma cells as compared to the TMZ-sensitive cell line LN229 and normal human astrocytes. At low concentrations, Ouabain selectively killed T98G cells. Knocking down the NKA α3 isoform sensitized T98G cells to TMZ and caused more cell death.

#### 3.1.4 Cell cycle arrest

p21^CIP1^ is a cyclin-dependent kinase inhibitor (CDK-I) that is regulated downstream of p53. When associated with Cdk1/2, it acts as an inhibitor of the cell cycle by blocking progression through the G1/S phase. Loss of p21^CIP1^ unleashes Cdk1 activity which causes chromosome instability. Besides promoting cell cycle progression Cdk1 has been described to play a role in cell cycle arrest, in particular at the G2/M checkpoint. Jing et al. (1994) aimed to study the mechanism of differentiation of human leukemia ML1 cells induced by Bufalin (Jing et al., 1994). The authors measured the effect of 10 nM Bufalin on cell growth, activities of several protein kinases, and cell cycle. The ML1 cell growth was inhibited significantly. Activities of PKC and PKA were inhibited whereas cdc2 kinase (=Cdk1) was increased early by Bufalin. Cell cycle changes became evident at 12 h after treatment of ML1 cells with Bufalin and the cells were preferentially arrested in the G2/M phase. Similarly, p21^CIP1^ may have dual activities according to the degree of DNA damage (Cmielová and Rezáčová, 2011; Xu et al., 2010; Wang et al., 2021; Kometiani et al., 2005; Tian et al., 2009).

#### 3.1.5 Inhibition of migration

Migration is a prerequisite for tumor metastasis and defines the aggressiveness of a tumor. Pongrakhananon et al. (2013) revealed that treatment with Ouabain at physiological concentrations (10–30 pM) is able to inhibit the migration of human lung cancer H292 cells (Pongrakhananon et al., 2013). The effects of Ouabain were found to be mediated through the suppression of migration regulatory proteins, such as focal adhesion kinase (FAK), ATP-dependent tyrosine kinase (Akt), and cell division cycle 42 (Cdc42). Moreover, anti-migratory Ouabain actions were mediated via a ROS-dependent mechanism because the addition of ROS scavengers (N-acetylcysteine and glutathione) could reverse the effect of Ouabain on cell migration. On the other hand, Ouabain was shown to decrease the cancer cell adhesion to endothelial cells. Another study confirmed this seemingly paradox effect (Liu et al., 2013; Ninsontia and Chanvorachote, 2014; Shin et al., 2015; Magpusao et al., 2015).

Ruanghirun et al. (2014) demonstrated that very low concentrations of Ouabain (0–10 pM) facilitate cancer cell detachment from the extracellular matrix in human lung H23 cancer cells, while having minimal effect on cell viability (Ruanghirun et al., 2014). Detachment of cancer cells is a pre-conditional process for metastasis. The detachment-inducing effect of Ouabain was found to be mediated through activation (!) of FAK and Akt pathways. This study is in striking contrast to the former cited one which saw a reduced FAK activity in response to Ouabain treatment. We discuss this contradiction below.

#### 3.1.6 Cell growth inhibition by downregulation of (the following listed mechanisms)

##### 3.1.6.1 NKA isoform α1

We will mention later, in the chapter about NKA isoforms, more studies dealing with the NKAα1 as a survival receptor. Here we will cite one of the most important studies. Tian et al. (2009) demonstrated that Ouabain-induced cell growth regulation is intrinsically coupled to and dictated by changes in cell surface expression of NKAα1 via the PI3K/Akt/mTOR pathway (Tian et al., 2009). While Ouabain increases the endocytosis of the NKA in one benign (LLC-PK1) as well as human breast (BT20), and prostate (DU145) cancer cell lines only in LLC-PK1 but not in BT20 and DU145 cells Ouabain stimulates the PI3K/Akt/mTOR pathway and consequently upregulates the expression of NKA. This upregulation is sufficient to replete the plasma membrane pool of NKAα1 and to stimulate cell proliferation in LLC-PK1 cells. On the contrary, in BT20 and DU145 cells Ouabain leads to a gradual depletion of NKAα1 at the cell surface and an increased expression of cell cycle inhibitor p21cip, which consequently inhibits cell proliferation. They observed that siRNA-mediated knockdown of NKAα1 is sufficient to induce the expression of p21cip and slow the proliferation also of the benign LLC-PK1 cells.

##### 3.1.6.2 HIF-1α

The hypoxia inducible factor (HIF-1α) plays a crucial role especially in GBM, where it promotes stem cell survival in hypoxic niches. Nigim et al. (2015) presented a new GBM model, MGG123, which was established from a recurrent human GBM. Orthotopic xenografting of stem-like MGG123 cells reproducibly generated lethal tumors that were characterized by hypervascularity and robust stem cell marker expression. Hypoxia enhanced HIF-1α expression in cultured MGG123 cells, which was suppressed by Digoxin or Ouabain (Nigim et al., 2015). *In vivo*, treatment of orthotopic MGG123 xenografts with Digoxin decreased HIF-1α expression as well as vascular endothelial growth factor (VEGF) mRNA levels within the tumors, and extended survival of mice bearing the aggressive MGG123 GBM. The effect of Ouabain was not checked in this *in vivo* model (Zhang et al., 2008; Cao et al., 2014; Yang et al., 2018).

##### 3.1.6.3 NF-κB

NF-κB is considered a pro-inflammatory and survival signaling pathway, activated by interleukin 1 (IL-1α) and tumor necrosis factor α (TNFα). In general, IL-8 mRNA induction is regulated mainly by NF-κB. Saito et al. (2019) examined the effects of Ouabain on spontaneous IL-8 and IL-1α secretion in the HSC3 oral squamous cell carcinoma cell line. IL-8 secretion was reduced whereas IL-1α secretion was increased by Ouabain. Further analysis revealed that Ouabain induced phosphorylation of the activator protein (AP)-1 components c-Jun and c-Fos but not of the nuclear factor kappa B (NF-κB) components p65 and p50. The inhibitory effect of Ouabain was correlated to a diminished nuclear translocation of the NF-κB p65 subunit (Saito et al., 2019). The authors concluded that Ouabain seemingly exerts opposing effects on the transcription factors NF-κB and AP-1 (Mijatovic et al., 2006a).

#### 3.1.7 Other novel anti-tumor mechanisms by Ouabain

##### 3.1.7.1 FGF-2

One if the less known activities of Ouabain is the suppression of the extracellular release of firoblast growth factor 2 (FGF-2) protein from malignant cells, which is not dependent on the classical ER Golgi secretory pathway but on NF-κB signaling. Wakisaka et al. (2002) demonstrated that this pathway is activated by the Latent Membrane Protein 1 (LMP1), the main EBV oncoprotein and suppressed by Ouabain (Wakisaka et al., 2002). In this context, it is interesting that LMP1 activates also HIF-α1, a pathway which is suppressed by Ouabain, so that an up-stream convergence of LMP1 and Ouabain pathways may be concluded (Wakisaka and Pagano, 2003). Remarkably, it was shown that LMP1 increased the release of exosomes enriched with FGF-2 and that NKA was redistributed to late endosomes indicating an active role of NKA activity in FGF-2 secretion (Ceccarelli et al., 2007). We may assume that here a general inhibitory effect of Ouabain on the creation and/or release of exosomes is revealed. This could have enormous implications for tumor metabolism/biology as exosomes carry a lot of growth factors, transmitters, hormones.

##### 3.1.7.2 Nucleolus disorganization

Mijatovic et al. (2008) used a new generated cardenolide UNBS1450 and showed that the compound (100 nM) significantly impaired the dynamics of the actin cytoskeleton. In addition to disorganizing the actin cytoskeleton, UNBS1450 provoked striking effects on nucleolar morphology, including fractionation, compaction, and formation of dark enlargements (Mijatovic et al., 2008). They concluded that primary actin cytoskeleton disorganization may lead to unprocessed RNA accumulation in nucleoli and/or to transcribed RNA sequestration due to impaired nucleo-cytoplasmic trafficking. To our knowledge there are so far no reports if Ouabain has the same effect.

##### 3.1.7.3 Anoikis

Normal epithelial cells undergo apoptosis upon detachment from the extracellular matrix, a process termed “anoikis.” However, malignant epithelial cells with metastatic potential resist anoikis and can survive in an anchorage-independent fashion. Resistance to anoikosis is one of the most important mechanisms to promote and facilitate distant metastases. To identify novel anoikis sensitizers in anoikis-resistant PPC-1 prostate adenocarcinoma cells, Simpson et al. (2009) screened a library of 2,000 off-patent drugs and natural products. They identified several members of the family of cardiac glycosides as anoikis sensitizers, including Ouabain, Peruvoside, Digoxin and Digitoxin (Simpson et al., 2009). Ouabain initiated anoikis through caspase-8 activation. In addition, Ouabain sensitized cells to anoikis by inhibiting the NKA and inducing hypoosmotic stress.

##### 3.1.7.4 Mitochondrial membrane potential

The loss of the mitochondrial membrane potential (MMP) is a hallmark of apotosis - opening of the mitochondrial permeability transition pore has been demonstrated to induce depolarization of the transmembrane potential (Δψ_m_), release of e.g., apoptosis-inducing factor (AIF) and loss of oxidative phosphorylation. Yin et al. (2009) found that the mitochondrial toxin rotenone caused a rapid mitochondrial membrane potential collapse in Jurkat cells followed by plasma cell membrane (PCM) depolarization (Yin et al., 2009). Rotenone-induced PCM depolarization occurred before apoptosis and correlated well with NKA impairment. The authors demonstrated that both PCM depolarization and NKA impairment induced by rotenone were regulated by mitochondrial H2O2 and downregulation of Bcl-2. Interestingly, NKA suppression by Ouabain greatly accelerated and enhanced mitochondrial toxins-induced cells apoptosis in Jurkat cells. They proposed that mitochondria-to-Na^+^/K^+^-ATPase impairment and PCM depolarization might represent a novel mechanism for mitochondria to amplify death signals in the initiation stage of apoptosis induced by mitochondrial toxins.

##### 3.1.7.5 Gap junctions

Gap junctions are molecular structures that enable communication between neighboring cells. It has been shown that gap junctional intercellular communication (GJIC) is significantly reduced in cancer cells compared to their normal counterparts. Serrano-Rubi et al. (2020) analyzed by means of dye transfer assays whether Ouabain (0,1–500 nM) affects GJIC in cervico-uterine (CasKi, SiHa and Hela), breast (MDA-MB-321 and MCF7), lung (A549), colon (SW480) and pancreas (HPAF-II) cancer cell lines. They found that Ouabain induces a statistically significant enhancement of GJIC in all of these cancer cell lines (Serrano-Rubi et al., 2020). Remarkably, the synthesis of new protein subunits is not required. C-SrC, ERK1/2 and ROCK-Rho are involved in mediating the signaling mechanisms.

##### 3.1.7.6 p53

p53 is the most important tumor suppressor protein and the most mutated gene in tumors. In normal cells, p53 protein binds to DNA, stimulating the production of p21 that interacts with cdk2. When p21 is in complex with cdk2 the cell cannot pass through to the next stage of cell division, i.e., causing cell cycle arrest. Mutated p53 has lost its ability to bind DNA. Wang et al. (2009) reports that in multiple human cancer cell lines the basal level of p53 protein is downregulated by Digoxin and Ouabain at nanomolar concentrations, independent of p53 status (wild-type/mutant) (Wang et al., 2009). They demonstrated that p53 reduction is triggered by activation of Src/mitogen-activated protein kinase (MAPK) signaling pathways upon CTS binding to the NKA and can be completely blocked by inhibitors of Src or MAP/ERK kinase. The authors concluded a novel effect of Ouabain considering mutant p53 acts as a driver oncogene in the gain-of function model.

##### 3.1.7.7 Unfolded protein response (UPR)

The unfolded protein response (UPR) is activated in response to an accumulation of unfolded proteins in the lumen of the endoplasmic reticulum to restore normal function of the cell by halting protein translation, degrading misfolded proteins or, after prolonged disruption, inducing apoptosis. Ozdemir et al. (2012) studied the possible effects of Ouabain on proliferation, apoptosis, and the unfolded protein response (Ozdemir et al., 2012). HepG2 cells were treated with Ouabain (0,75–750 nM) in the absence or presence of 10 mM 2-deoxyglucose (2-DG) for 48 h. They showed that Ouabain modulates the UPR transcription program and induces cell death in glucose-deprived tumor cells. Interestingly, they observed no cytotoxic effect of Ouabain in the presence of glucose.

##### 3.1.7.8 Senolysis

In addition to its anti-tumor effects, recent studies implicated Ouabain as a new senolytic agent. Senolytic drugs selectively eliminate senescent cells (Chang et al., 2024). Senescence is defined as a stable proliferation arrest mainly occurring as “stress response” in aged, damaged and preneoplastic cells. But even these cells have stopped dividing they remain metabolically active (Ark and Uddin, 2023). Their effects in the aging process and cancer development are mediated by secreting proinflammatory cytokines, growth factors, and proteases, collectively referred to as the senescence-associated secretory phenotype (SASP) (Jiang et al., 2024). With respect to cancer research senescence is especially fascinating–on the one hand it is a tumor suppressive process when induced by oncogenes (OIS) hereby limiting the proliferation of damaged cells, on the other hand senescent cells have a pre-malignant profile with in general hypomethylated DNA but localized hypermethylation especially of the promoter regions (Cps islands) of tumor suppressors (Cruickshanks et al., 2013). When these cells overcome the proliferation barrier they may initiate and/or promote tumorigenesis. Ouabain has been demonstrated to act as a highly effective senolytic drug in a variety of cancer cells (Hôte et al., 2021). The mechanism involved in its senolytic effects includes in between others the induction of the BH3-only pro-apoptotic protein.

### 3.2 Effects of (exogenous) Ouabain

#### 3.2.1 In vitro

##### 3.2.1.1 Anti-Cancer

###### 3.2.1.1.1 Prostate cancer

Huang et al. (2004) determined the therapeutic potential of cardiac glycosides in PC-3 androgen-independent prostate cancer cells and revealed two dose-dependent distinct modes of cytotoxic action (Huang et al., 2004). Both low and high concentrations of Ouabain induced an inhibition of Na^+^-K^+^ ATPase and a subsequent Ca^++^ influx into PC-3 cells. High concentrations of Ouabain (>10 nM) induced a significant and time-dependent loss of mitochondrial membrane potential (Δψ_m_), a sustained production of reactive oxygen species (ROS), and severe apoptotic reaction. Low concentrations of Ouabain (<10 nM) induced an increase of Par-4 (prostate apoptosis response 4) expression (Yeh et al., 2001; McConkey et al., 2000; Smith et al., 2001).

###### 3.2.1.1.2 Breast carcinoma

The following study is of special interest as it describes the involvement of Ouabain in estrogen metabolism. Busonero et al. (2020) mentioned that prolonged endocrine therapy often leads to ER-resistance and results in metastatic disease, for which a standardized effective therapy is still lacking. They report that Ouabain activates the cellular proteasome, resulting in ERα degradation, induces cell cycle blockade in the G2 phase, and triggers apoptosis (Busonero et al., 2020). Remarkably, these effects are independent of the inhibition of the Na^+^/K^+^-ATPase. The anti-proliferative effects of Ouabain and Digoxin occur also in diverse cancer models (i.e., tumor spheroids and xenografts). Interestingly, gene profiling analysis revealed that Ouabain downregulates the expression of genes related to endocrine therapy resistance (Chen et al., 2006; Winnicka et al., 2007; Bielawski et al., 2006; Khajah et al., 2018; Acconcia, 2022).

###### 3.2.1.1.3 Lung cancer

The study below is important because it reveals a potential new biomarker (STK11 mutation) in progressive lung cancer responding to CTS therapy. Kim et al. (2016) describes STK11 mutation as a major mediator of lung cancer progression. They report that targeting the Na^+^/K^+^-ATPase isoform α1 (NKA α1) is synthetic lethal with STK11 mutations in lung cancer. Digoxin, Digitoxin and Ouabain exhibited selective anticancer effects on STK11 mutant lung cancer cell lines (Kim et al., 2016). Increased cellular ROS production was associated with the STK11-specific efficacy of CTS. The authors claim that these results show that STK11 mutation is a novel biomarker for responsiveness to CTS. Inhibition of NKA α1 using CTS needs exploration as a targeted therapy for STK11 mutant lung cancer (Chanvorachote and Pongrakhananon, 2013; Trenti et al., 2014; Wang et al., 2012; Liu et al., 2013; Ninsontia and Chanvorachote, 2014; Shin et al., 2015; Pongrakhananon et al., 2013; Mijatovic et al., 2006b).

###### 3.2.1.1.4 Hepatocellular carcinoma

This study is of special interest as it revealed cardiotonic steroids as highly effective in aggressive hepatocellular carcinoma (HCC). Song et al. (2020) analyzed whether the spheroid model MCTS simulate *in vivo* tumor microenvironments. Through a high-throughput screening for HCC therapy using the MCTS model, inhibitors of Na^+^/K^+^-ATPase (Ouabain and Digoxin) were selected. Both suppressed cell growth and migration via inhibition of epithelial-mesenchymal transition (EMT) of HCC *in vivo* and *in vitro* (Xu et al., 2010; Xu et al., 2011; Fu et al., 2020).

This result is striking as other authors saw an increase of EMT in prostate cancer under Ouabain treatment (Banerjee et al., 2021). This discrepancy needs further evaluation.

###### 3.2.1.1.5 Gastric and esophageal cancer

As far as we know this is the first study showing an anti-cancer effect of Ouabain in gastric cancer. Chen et al. (2021) demonstrated that Ouabain (at 0–1,6 µM) decreased gastric adenocarcinoma (AGS) cell proliferation, cell viability, and motility (Chen et al., 2021). In addition, Ouabain inhibited AGS cell migration and invasion. Analyzing the signaling pathways the authors saw under Ouabain treatment reduced levels of proteins associated with PI3K/AKT and p38/MAPK pathways. In addition, at 48 h Ouabain decreased the expression of proteins involved in migration and metastasis such as N-cadherin, tissue inhibitor of metalloproteinases-1 (TIMP-1), urokinase-type plasminogen activator (c-uPA), and MMP-2.

###### 3.2.1.1.6 Biliary tract cancer

Also the following study is a first-time study in a highly aggressive cancer. Mayr et al. (2023) stresses that similar to HCC biliary tract cancer is a deadly disease with limited therapeutic options. They found that Ouabain has a strong cytotoxic effect with IC50 levels in the (low) nM-range, mainly by inducing apoptosis (Mayr et al., 2023). Interestingly, this effect was not associated with any change in the mRNA expression of the Na^+^/K^+^-ATPase α, β and fxyd subunits In addition, using a 3D cell culture model, they revealed that Ouabain disturbs spheroid growth and reduces the viability of biliary tract cancer cells within the tumor spheroids.

###### 3.2.1.1.7 Ovarial cancer

One preliminary study is dealing with the potential anti-tumor activity of CTS in Ovarial cancer. Chou et al. (2021) investigated the effects of CTS on proliferation, cytotoxicity and cell cycle of the ovarian cancer cell line (SKOV-3). Digoxin and Digitoxin at concentrations higher than IC50 (2.5 × 10^−7^ M/4.0 × 10^−7^ M) decreased cell proliferation of SKOV-3 cells (Chou et al., 2021). Interestingly, Ouabain showed a dual effect with inhibition at high doses (µM) and stimulation at low (pM) doses. The colony-formation ability was reduced after treatment with Digoxin and Digitoxin up to 10 days and both led to cell cycle arrest in G_0_/G_1_ phase within 24 h.

###### 3.2.1.1.8 Osteosarcoma

Osteosarcoma is the most common malignant bone tumor in children. The poor prognosis is due to high metastatic potential and resistance to current therapies. It is fascinating to see several studies demonstrating a potential benefit of Ouabain and other CTS. Delebinski et al. (2015) screened different herbal extracts for their anti-tumor potential in the osteosarcoma cell line 143B. They revealed that various steroid glycosides suppress cell proliferation in a concentration-dependent manner. Remarkably, apoptosis was induced by 17 of the 20 tested cardenolides and bufadienolides (Delebinski et al., 2015). Proscillaridin A and Ouabain revealed the strongest apoptotic induction, associated with breakdown of MMP and activation of caspases 8/9 (Chou et al., 2018; Guo et al., 2019; Shih et al., 2017; Yang JL. et al., 2021).

###### 3.2.1.1.9 Melanoma

Also melanoma is known to be insensitive to conventional chemotherapy and it remains a therapeutic challenge even with the emergence of checkpoint inhibitors. Here is one promising study with CTS. Wang et al. (2021) demonstrated that Ouabain strongly inhibits cell proliferation and triggers dramatic morphological changes in A375 melanoma cells (Wang et al., 2021). Ouabain induced significant apoptosis in A375 cells via upregulation of Bax and downregulation of Bcl-2. Moreover, Ouabain induced cell cycle arrest at G2/M phase in both A375 and SK-Mel-28 cells via upregulation of cyclin B1 and downregulation of cell division cycle protein 2 (cdc2) and cdc25c. Last but not least, Ouabain suppressed also the migration of A375 and SK-Mel-28 cells.

###### 3.2.1.1.10 Thyreoid cancer

Anaplastic thyroid carcinoma (ATC) is a rare, but aggressive, carcinoma derived from follicular cells with still a high mortality. Teixeira et al. (2022) aimed to evaluate Ouabain effects on 85-05C human anaplastic thyroid cells. Viability, cell death, cell cycle, colony formation and migratory ability were analyzed. The expression of (“positive”) differentiation and (“negative”) epithelial-to-mesenchymal transition (EMT) markers were also quantified in these cells. They showed that Ouabain, on the one hand, decreased the number of viable 8505C cells as well as cell migration (Teixeira et al., 2022). On the other hand, Ouabain decreased mRNA levels of Thyroid Transcription Factor-1 (TTF1) an important differentiation marker, and increased mRNA levels of N-cadherin, an EMT marker. The authors came to the important conclusion which needs to be evaluated in other tumors that Ouabain may have anti-proliferative and anti-migratory effects on 8505C cells, but seems to maintain an aggressive and undifferentiated profile of ATC. As mentioned above the data about the effects of Ouabain on EMT are contradictious and need clarification.

###### 3.2.1.1.11 Glioblastoma multifome

We will cite here a few studies because our group is focusing on the effects of Ouabain in Glioblastoma multiforme (GBM) the most aggressive brain tumor with still a very poor prognosis. Hsu et al. (2015) analyzed Epi-reevesioside F, a new cardiac glycoside, in T98G and U-87 glioblastoma cells, which was shown to be more potent than Ouabain (Hsu et al., 2015). However, both Epi-reevesioside F and Ouabain were ineffective in A172 cells, a glioblastoma cell line with low Na^+^/K^+^-ATPase α3 isoform expression. This underlines the importance of our hypothesized switch from NKAα1 to NKAα3 expression in endogenous tumor defense. Epi-reevesioside F induced cell cycle arrest at S and G2 phases and caspase-dependent apoptosis. Notably, Epi-reevesioside F caused cytosolic acidification that was highly correlated with the anti-proliferative activity. All these actions were correlated to inhibition of the PI3-kinase/Akt pathway. Ono et al. (2016) dealt with Glycoprotein non-metastatic melanoma protein B (GPNMB) which is involved in invasion and metastasis. High levels of GPNMB and Na^+^/K^+^-ATPase α1 subunits are associated with a poor prognosis in glioblastoma patients. It was revealed that GPNMB interacts with NKA α1 subunits to activate PI3K/Akt and MEK/ERK pathways. Interestingly, Ouabain suppressed the glioma growth induced by the injection of glioma cells in the transgenic mice overexpressing GPNMB and blocked the GPNMB-induced migration of glioma cells (Ono et al., 2016). Yang et al. (2018) elucidated the effect of Ouabain on U-87MG human glioma cell apoptosis and investigated the exact mechanism. Compared with the control group, Ouabain suppressed U-87MG cell survival, and attenuated cell motility in a dose-dependent manner (Yang et al., 2018). The downregulation of p-Akt, mTOR, p-mTOR and HIF-1α were observed following treatment with 2,5 and 25 μmol/L of Ouabain. The fact, that here relatively high doses were used, needs to be discussed. Weidemann et al. (2023) aimed to demonstrate a divergent effect of Ouabain on a TMZ-resistant (T98G) as compared to a TMZ-sensitive (LN229) GBM cell line. T98G cells showed a significant inhibition of cell migration and a significant depolarization of the plasma cell membrane potential (PCMP) at similar Ouabain concentrations with a strong inverse correlation (Weidemann et al., 2023). In contrast, LN229 cells did not respond to Ouabain in these assays at all. Similarly, only T98G but not LN229 cells revealed Bcl-2 downregulation at nanomolar Ouabain concentrations. This unique response to Ouabain is associated with a downregulation of pan-Akt in T98G cells 24 h after Ouabain (1.0 × 10^−6^ M) treatment. For the first time, we could show an anti-angiogenic effect of Ouabain on HUVEC cells which correlated strongly with the anti-migratory effect. In summary, we revealed that the TMZ-resistant T98G cell line as compared to the TMZ-sensitive LN229 cell line shows a high sensitivity towards Ouabain.

###### 3.2.1.1.12 Leukemia and lymphoma

Acute myeloid leukemia (AML) is an especially aggressive hematologic malignancy characterized by an accumulation of immature leukemic myeloblasts initiating from leukemic stem cells (LSC) which are, like in other tumor entities, responsible for chemotherapy resistance and relapse. Poohadsuan et al. (2023) tested the effects of Ouabain and other CTS on various human AML-derived cells with different maturation phenotypes. Regulatory mechanisms underlying cardiac glycoside-induced cytotoxicity were investigated and linked to cell cycle distribution and apoptotic machinery. Primitive AML cells containing CD34^+^ LSCs were very responsive to nanomolar concentrations of cardiac glycosides, with Ouabain showing the greatest efficiency (Poohadsuan et al., 2023). Ouabain preferentially induces caspase-dependent apoptosis in LSC, independent of its cell differentiation status. Interestingly, Mcl-1 and c-Myc were found to be the key apoptosis mediators that determined Ouabain sensitivity in AML cells. Specifically, Ouabain induces a rapid loss of Mcl-1 and c-Myc in LSC via an inhibition of Mcl-1 protein synthesis and an induction of c-Myc degradation (Feng et al., 2016; Zheng et al., 2021).

##### 3.2.1.2 Pro-cancer

Xu et al. (2009a) analyzed the lymphocytic leukemia Jhhan cells and megakaryocytic leukemia M07e cells which were incubated at different Ouabain (1 and 10 nmol) concentrations of for 24 h 1 nmol and 10 nmol Ouabain promoted proliferation of both Jhhan and M07e cells. Ouabain also increased the expression of Na^+^/K^+^-ATPase α1 at the cell surface (Xu et al., 2009a). Addition of either Src kinase inhibitor PP2 or MEK inhibitor PD98059 blocked the effects of Ouabain on cell proliferation. The authors concluded that Ouabain activates Src and ERK1/2 pathways and regulates the proliferation of leukemia cells. To mention that the same group (Wang et al., 2007) used a similar experimental design before with different resp. Opposite results (Wang et al., 2007). They also used the mekakaryocytic leukemic M07e (and Meg-01) and lymphocytic leukemia Jhan (and B95) cell lines and applied the same low Ouabain concentrations (1 and 10 nmol). Whereas proliferation of lymphocytic leukemia cells indeed was stimulated by low dose Ouabain and NKAα1 upregulated, the megakaryocytic leukemia cells were inhibited in cell growth and the NKAα1 was downregulated. The reason for these discrepancies in similar study designs is not clear, they need to be verified. All in all, very low Ouabain concentrations were used which are known to stimulate cell growth.

The next study is a rare demonstration of Ouabains low efficiency in malignant cells. Clifford and Kaplan (2013) investigated the effects of CTS compounds Ouabain, Digitoxin, and Bufalin on cell growth and survival in cell lines exhibiting the full spectrum of non-cancerous to malignant phenotypes. They showed that CTS inhibit membrane Na^+^/K^+^-ATPase activity equally well in all cell lines tested regardless of metastatic potential. In contrast, the cellular responses to the drugs are different in non-tumor and tumor cells (Clifford and Kaplan, 2013). Ouabain (100 or 500 nM) caused greater inhibition of proliferation and more extensive apoptosis in non-tumor breast cells compared to malignant cells. In tumor cells, the effects of Ouabain are accompanied by activation of anti-apoptotic ERK1/2. However, ERK1/2 or Src inhibition does not sensitize tumor cells to CTS cytotoxicity, suggesting that other mechanisms provide protection to the tumor cells. These results which are contradictory to many other results in the literature need further evaluation. We postulate that the *duration* of activation of e.g., ERK1/2 as well as Ouabain concentrations and NKA isoform patterns at the cell surface could each be crucial to direct cells either to enhanced or decreased cell proliferation. We will summarize this issue below in the discussion.

#### 3.2.2 In vivo

##### 3.2.2.1 Anti-Cancer

Here we cite some studies only shortly as they were mentioned already above in the *in vitro* part.

###### 3.2.2.1.1 Lung cancer

Mijatovic et al. (2006) starts from the observation that overexpression of the heat shock protein 70 (Hsp70) in NSCLC is (in between other factors) responsible for the failure of currently used chemotherapeutic drugs. They aimed to characterize *in vitro* and *in vivo* the antitumor effects of a new cardenolide (UNBS1450) on experimental human NSCLC. UNBS1450 is known as a potent source of *in vivo* antitumor activity in subcutaneous human NCI-H727 and orthotopic A549 xenografts in nude mice. They could demonstrate that UNBS1450 mediates the decrease of Hsp70 at both mRNA and protein levels (Mijatovic et al., 2006b). Kim et al. (2016) as mentioned above could demonstrate also *in vivo*, that clinically relevant doses of Digoxin decreased the growth of STK11 mutant xenografts compared to wild type STK11 xenografts (Kim et al., 2016).

###### 3.2.2.1.2 HCC

Yang S. et al. (2021) aimed to explore a novel strategy to potentiate the anti-HCC effects of Sorafenib to which many responders become insensitive after long-term treatment. They used HCC cell lines, siRNA and a tumor xenograft mouse model to determine the anti-HCC effects of Sorafenib in combination with Berbamine or other Na^+^/K^+^-ATPase ligands, i.e., CTS. They demonstrated that Ouabain synergizes with Sorafenib to inhibit HCC cells growth (Yang S. et al., 2021). Mechanistically, the Na^+^/K^+^-ATPase ligand Ouabain induces Src, EGFR, type I insulin-like growth factor receptor, ERK1/2 and p38MAPK phosphorylation in hepatocellular carcinoma cells. In contrast, Sorafenib inhibits the induction of Src, p38MAPK, EGFR and ERK1/2 phosphorylation by Ouabain. Importantly, combination of Sorafenib with Ouabain synergistically inhibits also Sorafenib-resistant HCC cells growth. Co-treatment of hepatocellular carcinoma cells with Ouabain and Sorafenib significantly induces cell death and significantly inhibits hepatocellular carcinoma xenografts growth *in vivo*.

###### 3.2.2.1.3 Melanoma

We cite below studies from da Silva et al. (2019) - they showed that *in vivo* Ouabain treatment reduced regulatory T cells in the spleen in both melanoma B16F10 and non-melanoma groups. Ouabain preserved the number and percentage of B lymphocytes in peripheral organs of melanoma-injected mice. Importantly, Melanoma-injected mice pre-treated with Ouabain survived longer (da Silva et al., 2019).

###### 3.2.2.1.4 Glioblastoma

We mentioned above the study of Nigim et al. (2015) who developed a new GBM model. Remarkably, the treatment of orthotopic MGG123 xenografts with Digoxin or Ouabain decreased HIF-1α expression as well as vascular endothelial growth factor (VEGF) mRNA levels within the tumors, and extended survival of mice bearing the aggressive MGG123 GBM (Nigim et al., 2015). The study by Ono et al. (2016) we already cited also demonstrates impressive *in vivo* effects of Ouabain. The interaction of GPNMB and Na+/K + -ATPase α isoforms was identified in the murine glioma model and in the tumors of glioblastoma patients. Ouabain suppressed glioma growth induced by the injection of glioma cells in the transgenic mice overexpressing GPNMB and blocked the GPNMB-induced migration of glioma cells (Ono et al., 2016).

###### 3.2.2.1.5 Lymphoma and leukemia

Zhang et al. (2008) screened a library of drugs that are in clinical trials as potential inhibitors of hypoxia-inducible factor 1 (HIF-1α). Eleven of 20 drugs were cardiac glycosides, including digoxin, ouabain, and proscillaridin A, which inhibited HIF-1α protein synthesis and expression of HIF-1α target genes in cancer cells. They showed that Digoxin administration increased latency and decreased growth of tumor xenografts, whereas treatment of established tumors resulted in growth arrest within 1 week (Zhang et al., 2008). The study from da Silva et al. (2019) we will cite below more in detail–interestingly, he saw only *in vivo* (mice), not *in vitro*, a reduction of number of CD4^+^ T lymphocytes, especially Tregs (da Silva et al., 2019).

##### 3.2.2.2 Pro-cancer

Remarkably, in our database research over a span of 50 years (!) we found only this recent work. Yang et al. (2023) described that endogenous ouabain (EO) is elevated in patients with NSCLC and closely related to tumor pathological stage, metastasis and survival (Yang et al., 2023). They revealed that EO increases PD-L1 transcription, however, the EO receptor Na^+^/K^+^-ATPase α1 interacts with PD-L1 to trigger the endocytic degradation of PD-L1. In light of these seemingly contradictory results the authors claim to have discovered the mechanism whereby EO cooperates with NKA α1 to finely control PD-L1 expression and dampen tumoral immunity. They concluded that the NKA α1/EO signaling facilitates immune escape in lung cancer. This challenging theory we will comment in the discussion.

### 3.3 Effects of exogenous Ouabain on the immune system

#### 3.3.1 Tumor suppressing

There are abundant data about the interaction between Ouabain and the immune system. We refer to some reviews (Rodrigues-Mascarenhas et al., 2009; Cavalcante-Silva et al., 2017; Leite et al., 2022) and will cite here only a few *in vivo* studies dealing with the influence of Ouabain on the altered number and function of immune cells.

Shih et al. (2019) developed WEHI-3 cell generated leukemia mice and treated them by oral Ouabain at 0, 0.75, 1.5 and 3 mg/kg for 15 days. Ouabain decreased liver and spleen weights, B- and T-cell proliferation at all three doses treatment and increased CD19 cells at 3 mg/kg treatment compared with positive control. Furthermore, Ouabain increased macrophage phagocytosis from peripheral blood mononuclear cells at all three doses and increased NK cell activities (Shih et al., 2019). Another study saw that Ouabain seems to target specifically regulatory T (Treg) cells. This is of special importance because Treg cells–an immunosuppressive subset of CD4^+^ T cells primarily responsible for self-tolerance - can suppress anticancer immunity, thereby hindering protective immunosurveillance of malignancies thus promoting tumor development and progression. Da Silva et al. (2019) aimed to show the effects of Ouabain on peripheral and spleen T lymphocytes. Mice were injected i. p. for 3 consecutive days with 0.56 mg/kg of Ouabain. They saw that Ouabain significantly reduced the number of CD4^+^ T lymphocytes in the spleen, especially regulatory T cells. Interestingly, *in vitro*, Ouabain did not inhibit the proliferation of CD4+T lymphocytes and was not able to induce the apoptosis of CD4^+^ and/or Tregs. Secretion of IL-2 by activated T lymphocytes was decreased by Ouabain, explaining at least in part the reduction of Tregs, since this cytokine is involved in the peripheral conversion and maintenance of Tregs (Da Silva et al., 2019).

There are a number of studies gaining interest about the role of CTS/Ouabain in Immunogenic Cell Death (ICD). Some chemotherapeutic compounds, anthracyclines and Oxaliplatin, induce a type of cell death that is immunogenic, i.e., transforming the patient’s dying cancer cells into a vaccine that stimulates antitumor immune responses. Menger et al. (2012) identified CTS as exceptionally efficient inducers of immunogenic cell death by means of a fluorescence microscopy platform. This effect was associated with the inhibition of the plasma membrane Na^+^-/K^+^-ATPase. Noteworthy, CTS exacerbated the antineoplastic effects of DNA-damaging agents in immune-competent but not immune-deficient mice. Moreover, cancer cells receiving a combination of chemotherapy plus CTS could vaccinate syngeneic mice against a subsequent challenge with living cells of the same type (Menger et al., 2012). Retrospective clinical analyses revealed that the administration of Digoxin during chemotherapy had a positive impact on overall survival in cohorts of breast, colorectal, head and neck, and hepatocellular carcinoma patients, especially when they were treated with agents other than anthracyclines and Oxaliplatin. Xiang et al., 2020 points out that most of the anticancer drugs cause non-immunogenic cell death. A serious problem is the persistence and expansion of treatment-resistant clones. They report a combination strategy that applies simultaneously non-immunogenic cell death inducer Cisplatin and Digoxin to treat primary tumors and convert the tumor cells into vaccines that enables a long-lasting immune response against residual tumors to prevent tumor recurrence and metastasis (Xiang et al., 2020). They revealed that complementary mechanisms induced potent immunogenic cell death that promotes dendritic cell maturation and activates CD8^+^ T cell responses. In established tumor models, Cisplatin and Digoxin combinations completely eradicate tumors with no residual cancer cells remaining.

Škubník et al. (2021b) aimed to summarize current knowledge in the field of immunomodulatory properties of CTS and emphasized the large area of potential clinical use of these compounds. He stressed the strong connection of CTS to immunogenic cell death. Moreover, CTS exert various immunomodulatory effects, mainly by suppressing the activity of T-helper cells or modulating transcription of many immune response genes by inhibiting nuclear factor- κB (Škubník et al., 2021b).

Shandell et al. (2022) points out that little are known about the effects of the ionic tumor microenvironment on immune checkpoint expression and function. They describe a mechanistic link between Na^+^/K^+^-ATPase inhibition and activity of the immune checkpoint protein indoleamine-pyrrole 2′,3′-dioxygenase 1 (IDO1). As they described earlier IDO1 was necessary and sufficient for production of kynurenine, a downstream tryptophan metabolite, in cancer cells. Metabolites of the IDO1-kynurenine pathway are known to activate PI3K-Akt signaling in neoplastic epithelium and promote cellular proliferation. In a spectrophotometric screening assay of 31 ion transport-targeting compounds it was revealed that Ouabain and Digoxin inhibited kynurenine production at concentrations that did not affect cell survival. NKA inhibition by Ouabain and Digoxin resulted in increased intracellular Na^+^ levels and downregulation of IDO1 mRNA and protein levels, which was consistent with the reduction in kynurenine levels (Shandell et al., 2022). NKAα1 knockdown significantly enhanced the effect of cardiac glycosides on IDO1 expression and kynurenine production. Mechanistically, they showed that cardiac glycoside treatment resulted in curtailing the length of phosphorylation-mediated stabilization of STAT1, a transcriptional regulator of IDO1 expression, an effect enhanced by NKA α1 knockdown.

#### 3.3.2 Tumor promoting

Here again it is the above mentioned study from Yang et al. (2023) who claims a negative impact of EO on the immune system by up-regulating the checkpoint protein PD-L1. As mentioned before we will deal with this important study in the discussion.

### 3.4 Expression and activity of NKA isoforms in tumors

#### 3.4.1 NKA α1 and NKA α3

Tian et al. (2009) showed in an elegant study that changes in the expression of Na^+^/K^+^-ATPase at the plasma cell membrane dictate the growth regulatory effects of Ouabain on cells via the PI3K/Akt/mTOR pathway (Tian et al., 2009). Ouabain increases the endocytosis and degradation of NKA α1 in benign LLC-PK1, human breast (BT20) and prostate (DU145) cancer cells. However, only in LLC-PK1 but not in BT20 and DU145 cells Ouabain stimulates the PI3K/Akt/mTOR pathway and consequently upregulates the expression of Na^+^/K^+^-ATPase. This upregulation is sufficient to replete the plasma membrane pool of NKAα1 and to stimulate cell proliferation in LLC-PK1 cells. On the other hand, in BT20 and DU145 cancer cells Ouabain causes a gradual depletion of NKAα1 and an increased expression of cell cycle inhibitor p21cip1, which consequently inhibits cell proliferation. Consistently, they observed that small interfering RNA-mediated knockdown of NKAα1 is sufficient to induce the expression of p21cip1 and slow the proliferation also of benign LLC-PK1 cells. They demonstrated that both Src and caveolin-1 are required for Ouabain-induced activation of PI3K/Akt and upregulation of Na^+^/K^+^-ATPase. Furthermore, inhibition of the PI3K/Akt/mTOR pathway by rapamycin completely blocks ouabain-induced expression of NKAα1 and converts Ouabain-induced growth stimulation to growth inhibition in LLC-PK1 cells.

Xu et al. (2010) also stressed the important role of the Na^+^/K^+^-ATPase α1 subunit in malignant cell ion transport, metabolism, migration and signal transduction. Using Ouabain and NKA α1 subunit siRNA they have evaluated the effects of inhibiting NKAα1 in human HepG2 cells. First of all, they showed that the expression of the NKA α1 subunit is higher in HCC tissues (including human HCC lines HepG2, SMMC-7721 and Bel-7402) than in normal liver tissues. Interestingly, Ouabain and NKA α1 siRNA could inhibit HepG2 cell proliferation (Xu et al., 2010). Moreover, Ouabain was able to induce HepG2 cell apoptosis via intracellular Ca^++^ and ROS increase and generate S phase arrest by decreasing the CyclinA1/CDK2/proliferating cell nuclear antigen (PCNA) complex and increasing the expression of cyclin-dependent kinase inhibitor 1A (p21cip1). Remarkably, NKAα1 siRNA could enhance all these anti-cancer effects of Ouabain.

The following study deals with the loss of NKAα1 at the tumor cell membrane and its correlation with aggressiveness. It may explain the negative impact of EO/NKA interaction in cancer as claimed by the Chinese group. Banerjee et al. (2021) observed that the surface expression of NKAα1 is significantly reduced in primary prostate tumors and further decreased in bone metastatic lesions. They showed that the *loss* of cell surface expression of NKAα1 induces epithelial-mesenchymal transition (EMT) and promotes metastatic potential and tumor growth of prostate cancer (PCa) by decreasing the expression of E-cadherin and increasing c-Myc expression via the activation of Src/FAK pathways (Banerjee et al., 2021). They propose that the reduced surface expression of NKAα1 in PCa is due to increased (complete?) endocytosis through the activation of the NKA/Src receptor complex. Using a high-throughput NKA ligand-screening platform, the authors have discovered MB5 as an inverse agonist of the NKA/Src receptor complex, capable of blocking the endocytosis of NKA. MB5 treatment increased NKA expression and E-cadherin in PCa cells, which reversed EMT and consequently decreased the invasion and cell growth. This discrepancy with other studies will be evaluated in the discussion.

Another important study shall be cited dealing with the changes in NKA (isoform) expression with increasing malignancy, again with a *loss* of NKA expression in highly aggressive cancers.

Salyer et al. (2013) treated four breast cancer cell lines (MCF-7, T47D, MDA-MB453, and MDA-MB231) and a non-cancerous breast cell line (MCF-10A) with Ouabain and measured cell proliferation. Ouabain (1 μM) decreased cell proliferation in all cell lines except MDA-MB453. Western blot of NKA α and β subunits showed α1, α3, and β1 expression in all cell lines except MDA-MB453 where NKA (α β) protein and mRNA were completely absent (Salyer et al., 2013). Potassium uptake, measured as +86Rb flux, was significantly higher in MDA-MB453 cells compared to MCF-10A cells but not due to NKA activity but an increase in voltage-gated potassium channel (KCNQ2) expression in MDA-MB453. Inhibition of KCNQ2 prevented cell growth and +86Rb uptake in MDA-MB453 cells but not in MCF-10A cells. All cancer cells had significantly higher vacuolar H-ATPase (V-ATPase) activity than MCF-10A cells. Inhibition of V-ATPase decreased +86Rb uptake and intracellular potassium in MDA-MB453 cells but not in MCF-10A cells.

Shibuya et al. (2010) also investigated the clinical significance of NKA α1-, α2-and α 3-isoform expression in hepatocellular carcinoma (HCC). Interestingly and somewhat contrary to the findings of Xu et al. (2010) the expression of NKA α3 protein in HCC tissues were significantly higher than in the accompanying non-tumor tissues, whereas no increased expression of NKA α1 and α2 proteins were observed in HCC compared to non-tumor tissues. Also remarkable is their observation that the Ouabain-sensitive NKA activities in the membrane fraction from HCC tissue were significantly higher than those from non-tumor tissues. The NKA activity was positively and significantly correlated with the protein expression level of the NKA α3 isoform (Shibuya et al., 2010).

Another study saw similar results, with a seemingly upregulation of the NKA α3 isoform in cancer tissues. Sakai et al. (2004) investigated the expression levels of Na^+^/K^+^-ATPase α-isoforms and their ATPase activities in human colorectal cancer tissue and the surrounding normal mucosa. A markedly (81 ± 5%) decrease in the protein expression of NKA α1-isoform was observed in all sampled cancer tissues compared with the normal mucosae (Sakai et al., 2004). Similar to Shibuya et al. (2010) the expression of the α3-isoform protein in the cancer tissue was higher than in the normal mucosa. The reason for these discrepancies is not clear. But there is a probability that endogenous Ouabain is modifying the NKA expression in favor of a better tumor defense mechanism, i.e., by up-regulating NKAα3 it renders the tumor cell more sensitive to apoptosis by Ouabain.

Lin et al. (2008) stressed the importance of the ratio of NKA α1/NKA α3 isoform expression at the plasma cell membrane. They chose twelve human tumor cell lines as well as cell culture models of human glioma HF U251 and U251 cells to examine factors of human tumor cell sensitivity to cardiac glycosides. They revealed that high expression of NKA α1 isoform in the presence of low NKA α3 expression correlated with increased resistance to inhibition of cell proliferation by cardiac glycosides such as Oleandrin, Ouabain and Bufalin (Lin et al., 2008). Interestingly, increased expression of NKA α1 is associated with increased cellular levels of glutathione resulting in diminished release of cytochrome c and caspase activation. Additionally, an increased colony-forming ability was noted in cells with high levels of Na/K-ATPase α1 expression. The authors suggested (similar to Mijatovic et al.) that the Na+/K+ -ATPase α1 isoform may be actively involved in tumor growth and cell survival.

Xiao et al. (2017) examined the effects of Ouabain on the viability and induction of cellular death of OS-RC-2 renal cancer cells as well as the levels of Ca^2+^ and reactive oxygen species. Moreover, the expression profile of the different Na^+^/K^+^-ATPase isoforms in NCI-H446 small cell lung cancer cells was determined. Interestingly, expression of the NKA α_3_ but not the NKA α_1_ isoform was associated with enhanced Ouabain sensitivity (Xiao et al., 2017). Ouabain inhibited cancer cell proliferation and induced apoptosis while no significant difference in the expression of NKA α_1_ and α_3_ isoforms was detected following 48 h of Ouabain treatment.

Numata et al. (2025) showed in a recent study that in Circulating Cancer Cells (CCC) isolated from gastric cancer patients, NKA α3 was predominantly localized in the plasma membrane but it moved to the cytoplasm when the CCCs were attached. They suggest that NKA α3 plays an essential role in the survival of CCCs in gastric cancer, and that digoxin enhances anoikis in detached (metastatic) gastric cancer cells by inhibiting the NKA α3 translocation from cytoplasm back to the plasma membrane, thereby reducing CCCs. They concluded that targeting NKA α3 may be a promising therapeutic strategy against CCC survival (Numata et al., 2025).

Another study points to the NaKA α3 as a favorable target in cancer treatment. Lindholm et al. (2022) used 3 pancreatic cancer cell lines, AsPC-1, Panc-1 and CFPAC-1 to investigate the anti-tumor effects of digitoxin in relation to the expression of the NKA subunits ATP1A1 (NKAα1) and ATP1A3 (NKA α3). Interestingly, digitoxin affected only the transcriptional but not the translational expression of the *ATP1A1* and *ATP1A3* subunits. In Panc-1 cells, *ATP1A3* gene expression was negatively associated with the digitoxin concentration (25–100 nM). In the other 2 cell lines, the expression of the *ATP1A1* gene increased in the cells treated with the high digitoxin concentration. The protein expression of ATP1A1 and ATP1A3 was not altered with digitoxin treatment. The basal protein expression of ATP1A1 was high in the AsPC-1 and CFPAC-1 cells, in contrast to the basal expression of ATP1A3, which was higher in the Panc-1 cells. In summary, the study demonstrates that the high expression of ATP1A3 renders pancreatic cancer cells more susceptible to digitoxin-induced cell death but also indicates that CTS down- or upregulate the gene expression of NKA isoforms (Lindholm et al., 2022).

It would be important to analyze why the transcriptional changes in NKA expression induced by CTS are not seen on the translational level (in case, this is an universal observed phenomenon).

Finally, we want to cite a very interesting study dealing also with an increased Ouabain sensitivity correlated with NKA α3 expression. Fujii et al. (2022) points out that the Glucose transporter GLUT1 plays a primary role in the glucose metabolism of cancer cells. They found that Ouabain, Oleandrin and Digoxin decreased GLUT1 expression in the plasma cell membrane of human cancer cells (liver cancer HepG2, colon cancer HT-29, gastric cancer MKN45 and oral cancer KB cells). The effective concentration of Ouabain was lower than that for inhibiting the activity of Na^+^/K^+^ -ATPase α1 isoform in the plasma cell membrane. The CTS also inhibited [(Newman et al., 2008) H]2-deoxy- d-glucose uptake, lactate secretion, and proliferation of the cancer cells. Remarkably, they revealed that in intracellular vesicles of human cancer cells, the Na^+^/K^+^ -ATPase α3 isoform is abnormally expressed (Fujii et al., 2022). A low concentration of Ouabain inhibited the activity of this vesicular NKA α3. Knockdown of NKA α3 but not NKA α1 significantly inhibited the Ouabain-dependent decrease in GLUT1 expression in HepG2 cells. Interestingly, all CTS decreased GLUT1 expression in dRLh-84 cells which were expressing the NKA α3 isoform at the plasma cell membrane. This would be a new mechanism by which endogenous Ouabain is able to suppress tumorigenesis.

#### 3.4.2 NKA activity

Here we cite only a few studies dealing with changes in NKA activity in malignant transformation. Already Kaplan (1978) described the leakage theory in pre-cancerous cells (Kaplan, 1978). For reasons not yet fully understood the transforming cells have an increased permeability of their cell membrane resulting in passive Na^+^ influx and K^+^ efflux. To compensate especially for the loss of potassium the NKA becomes hyperactive and hence is providing the tumor cell with nutrition (glucose) necessary for the aberrant increased tumor metabolism.

Gonta-Grabriec et al. (1986) observed that before thymoma could be discerned morphologically the activity of the Na^+^/K^+^-ATPase altered. In both, spontaneous and radiation-induced thymomas, +86Rb uptake, ATP hydrolysis and 3H-Ouabain binding per cell were higher than in normal thymuses. These changes correlated highly with cAMP content and 3H-thymidine incorporation, indicators of the increased proliferative activity typical for a pre-leukemic period (Gonta-Grabiec et al., 1986).

Michaels et al. (2024) departs from the observation that upregulated glycolysis in cancer cells results from increased demand for adenosine triphosphate (ATP) however it is unknown what this extra ATP turnover is used for. The authors hypothesized that one important reason for the increased glycolytic flux in cancer cells is the ATP demand of Na^+^/K^+^-ATPase due to altered sodium ion homeostasis in cancer cells (Michaels et al., 2024). Measurements of [Na^+^]_i_ and glycolytic flux were performed in three human breast cancer cells (MDA-MB-231, HCC 1954, MCF-7), in murine breast cancer cells (4T1), and control human epithelial cells MCF-10A, at baseline and after NKA inhibition with Ouabain. They revealed that basic intracellular [Na^+^]_i_ was elevated in human and murine breast cancer cells compared to control MCF-10A cells. This could correspond with the above mentioned leakage theory. Acute inhibition of NKA by Ouabain resulted in (further?) elevated [Na^+^]_i_ and inhibition of glycolytic flux in all three human cancer cells which are Ouabain-sensitive (but not in the murine cells which are Ouabain-resistant). Imitating the status of a pre-cancerous cell (“leakage”) they induced permeabilization of the cell membrane with gramicidin-A which led to an increase of [Na^+^]_i_ in MDA-MB-231 and 4T1 cells and a Na^+^-dependent increase in glycolytic flux (assumingly by consecutive increased NKA activity). Both these phenomenon again were attenuated by Ouabain in the human cells but not in the murine cells.

### 3.5 Ouabain dose-dependent effects on malignant (vs. benign) cells

The following section cites only a few of many studies which compare the effects of Ouabain on malignant as compared to non-malignant neighboring tissues. For more a more detailed summary we point to our review (Weidemann, 2012). All studies deal here with exogenous Ouabain.

Harich et al. (2023) used the SK-BR-3 breast cancer cell line, mesenchymal stem cells (MSCs), and tumor-associated fibroblasts (TAFs) *in vitro* to determine the effects of Ouabain exposure on these cellular types. In summary, they found multiple effects of Ouabain mainly on tumor cells, in a dose-dependent manner, while the TAFs and their normal counterparts were not significantly influenced. After exposure to Ouabain, the SK-BR-3 cells changed their morphologic appearance, decreased the expression of immunophenotypic markers (CD29, Her2, VEGF). Moreover, their proliferation rate was significantly decreased (Ki67 index) and the cells were blocked in the G_0_ phase of the cell cycle and suffered apoptosis/necrosis (Harich et al., 2023). These data were correlated with the changing expressions of α and β NKA isoforms in tumor cells, resulting in decreased ability to adhere to the VCAM-1 substrate in functional flow chamber studies. The group of Winnicka/Bielawski dealt in many studies with the duality of CTS actions in malignant as well as in benign cells. Winnicka et al. (2007) observed reduced cell viability in the human breast cancer cell line (MDA- MB-231) after applying Ouabain, Digoxin and Proscillaridin A in nmol ranges. They confirmed that cardenolides induce apoptosis in MDA-MB-231 cells by increasing free calcium concentration and by activating caspase-3. Notably, they revealed marked differences in the potency, with Proscillaridin A being the most active (IC50 48 ± 2 nM), followed by Digoxin (IC50 124 ± 2 nM) and Ouabain (IC50 142 ± 2 nM). All these concentrations are markedly lower than those needed to induce apoptosis in fibroblasts (Winnicka et al., 2007). Later, Winnicka et al. (2010) showed that low concentrations (30 nM) of Ouabain, Digoxin and Proscillaridin A can activate proliferation of human fibroblasts by increasing the level of phosphorylated extracellular signal-regulated kinases (p-ERK 1/2). Ouabain, Digoxin and Proscillaridin A only at a relatively high concentration of 300 nM increased intracellular Ca^++^ concentration, activated caspase-3 and induced apoptosis in human fibroblasts (Winnicka et al., 2010). Another study is an example that also in one and the same malignant cell line Ouabain can have dual activities in a dose-dependent way. Cuozzo et al. (2012) analyzed the pro-death and pro-survival properties of Ouabain in the human lymphoma derived cell line U937. He observed a dose-dependent dual action of Ouabain. Whereas high doses of Ouabain (>500 nM) caused ROS generation, elevation of Ca^++^ and death of lymphoma derived U937 cells lower doses of Ouabain (<100 nM) activated a survival pathway involving the Na^+^/Ca^++^-exchanger (NCX). Also p38 MAPK plays a pro-survival role, however, the activation of this MAPK does not appear to depend on NCX (Cuozzo et al., 2012).

### 3.6 Src-PI3K/Akt and ERK1/2 activation

We will cite in this section a few studies dealing with the role of ouabain-activated signaling pathways in tumor defense mechanisms, a somehow paradox phenomenon which will be discussed later in detail. Kometiani et al. (2005) explored the mechanism of the growth inhibitory effects of Ouabain on the estrogen receptor-negative human breast cancer cell line MDA-MB-43. Ouabain concentrations (<100 nM) had no effect on cell viability but inhibited proliferation. Their data suggest that Ouabain-induced transactivation of Src/EGFR by Na^+^/K^+^-ATPase leads to a transient and then a sustained activation of ERK1/2, followed by an increase in cell cycle inhibitor p21Cip1, resulting finally in growth arrest (Kometiani et al., 2005). Remarkably, Digoxin and Digitoxin concentrations close to or at the therapeutic plasma levels had effects on proliferation and ERK1/2 similar to those of Ouabain, supporting the potential value of CTS drugs for the treatment of breast cancer. The following author also points to a ‘switch’ from activation of a signaling pathway to its inhibition under prolonged treatment with CTS. Li et al. (2009) demonstrated that Bufalin inhibited the proliferation of gastric cancer MGC803 cells in a dose- and time-dependent manner. At low concentrations (20 nmol/L), Bufalin induced M-phase cell cycle arrest, whereas at a higher concentrations (80 nmol/L) it induced apoptosis via an increased Bax/Bcl-2 ratio and activated caspase-3 (Li et al., 2009). Remarkably, Bufalin transiently activated the PI3K/Akt signaling pathway and then inhibited it completely. A combination of Bufalin and LY294002, a PI3K-specific inhibitor, enhanced apoptosis, but PD98059, an ERK-specific inhibitor, had no significant effect on Bufalin-induced apoptosis.

The next study links the above cited important NKA α-isoform pattern on the cancer cell surface to the activation modus of ERK1/2. Karpova et al. (2010) worked with SK-N-AS human neuroblastoma cells, which co-express the α1 and α3 isoforms of the NKA. They silenced either the NKA α1 or NKA α3 isoform by means of transfection with siRNA and investigated cell survival and the cellular response to Ouabain. They observed that both α isoforms are essential for cell survival. In the presence of both NKA α isoforms, Ouabain causes sustained activation of ERK1/2. This activation is not affected when NKA α1 is silenced; however, when NKA α3 is silenced, Ouabain-induced activation of ERK1/2 does not occur, even at high Ouabain (1 μm) concentrations (Karpova et al., 2010). Thus, Ouabain-induced Erk1/2 activation is mediated in SK-N-AS cells only by NKA α3 and NKA α1 does not participate in this event (see: discussion).

Both of the two following studies focus on the role of Ouabain-activated signaling pathways in the induction of autophagy in lung cancer cells. It remains to be validated whether this is a manifestation of a basic feature specific for NSCLC. Trenti et al. (2014) examined the anticancer effect of Ouabain on non-small cell lung cancer cells lines A549 and H1975. Ouabain inhibited cell proliferation and induced cell death in a concentration-dependent manner (Trenti et al., 2014). Cell death was caspase-independent and showed classical patterns of autophagic cell death: conversion of LC3-I to LC3-II and increase of autophagic flux. The authors showed that Ouabain reduced Bcl-2 protein levels with no change in the expression of the autophagic protein Beclin 1. Early signaling events of Ouabain were ERK1/2 and JNK activation, however only JNK inhibition were able to prevent Bcl-2 decrease, conversion of LC3-I to LC3-II and cell death. They concluded that JNK activation by Ouabain leads to a decrease of Bcl-2 levels, resulting in disruption of the inhibitory interaction of Bcl-2 with Beclin1 that promotes autophagy.

Wang et al. (2015) analyzed the up-stream role of Src in the ERK1/2 signaling pathway as well as autophagic cell death induced by either Digoxin or Ouabain in A549 and H460 NSCLC cells. Src is significantly activated simultaneously with MEK1/2 and ERK1/2 activation upon CTS treatment (Wang et al., 2015). Src inhibitor PP2 as well as knockdown of Src with siRNA could block CTS induced MEK1/2 and ERK1/2 phosphorylation, as well as autophagic phenotypes in the cells. Moreover, increased levels of intracellular ROS are found to be involved in Src mediated autophagy. The authors hereby provide evidence that Src mediates MEK1/2 and ERK1/2 pathway as well as ROS generation, and regulates autophagic cell death induced by the cardiac glycosides. Here we want to cite one study with Src inactivation resulting in reduced lung metastasis. Shin et al. (2015) used the same lung cancer A549 cell line exploring the molecular mechanism by which Ouabain exerts anticancer effects. They found a downregulation of p-ezrin, a protein associated with pulmonary cancer metastasis in a dose-dependent manner (Shin et al., 2015). Furthermore, Western blot revealed the Ouabain-mediated downregulation of p-Src (Y416), p-FAK (Y925), p-paxillin (Y118), p130CAS, and Na^+^/K^+^-ATPase isoform α1 that all have been shown to be involved in the migration of cancer cells. Ouabain as well as Src inhibitor PP2 indeed inhibited cell migration.

The authors concluded that the anti-migratory effects of Ouabain on A549 lung cancer cells appear to be related to its ability to regulate and inactivate Src-to-ezrin signaling. This is in striking contrast to the above mentioned Src activation during the induction of autophagic cell death in lung cancer by CTS and may be explained by time and dose-dependent treatment differences.

Yan et al. (2015) investigated the molecular mechanism involved in Ouabain-induced ROS generation and cell apoptosis on human U373MG and U87MG glioma cells. Ouabain induced in both glioblastoma cells apoptosis and increased ROS generation. Clearance of ROS by three different ROS scavengers partly reversed the effect of Ouabain on cell apoptosis. Ouabain-induced ROS generation was not regulated by calcium overload but by p66Shc phosphorylation. Ouabain treatment increased p66Shc (Ser36) phosphorylation (Yan et al., 2015). Knockdown of p66Shc by siRNA significantly inhibited ROS generations in response to Ouabain. The p66Shc phosphorylation was mediated through the activated Src/Ras/ERK1/2 signal pathway.

The following studies are dealing with pro-cancer effects of Ouabain via ERK1/2 activation. The first study from Clifford and Kaplan (2013) which we cited above (see: “pro-cancer section) revealed not only ERK1/2 activation in tumor cells with reduced apoptosis upon Ouabain treatment but also a higher sensitivity of non-tumor cells towards CTS as compared to tumor cells (Clifford and Kaplan, 2013). The second pro-cancer study suggests similar to Karpova et al. a link between the NKA α isoform pattern and ERK1/2 activation. De Souza et al. (2014) used Caco-2 cells, a well-established human colorectal cancer model that does not exhibit caveolae. They demonstrated that Ouabain treatment resulted in a reduction of both the NKA α1 and NKA β1 protein and redistribution of the adherens junction (AJ) proteins E-cadherin and β-catenin. Furthermore, Ouabain increased claudin-3 protein levels, impaired the tight junction (TJ) barrier function and increased cell viability and proliferation during the early stages of treatment (de Souza et al., 2014). Additionally, the Ouabain-induced events were dependent on the (caveolae-independent) activation of ERK1/2 signaling. The authors concluded that α1 and β1 Na/K-ATPase downregulation and ERK1/2 activation induced by Ouabain are interlinked events that play an important role during cell-cell adhesion loss, which is an important step during tumor progression of colorectal carcinomas. The role of cell adhesion loss is in itself controversial in tumorigenesis und progression and needs further evaluation.

### 3.7 Metabolic effects

It is well known that the tumor cells use the Warburg effect for survival. The Warburg effect is defined as an increase in the rate of glucose uptake and preferential production of lactate, even in the presence of oxygen. The above cited study from Michaels et al. (2024) came to the conclusion that in breast cancer cells glycolytic flux correlates with Na^+^-driven NKA hyperactivity hereby “providing evidence for the centrality of the [Na^+^]i-NKA nexus in the mechanistic basis of the Warburg effect” (Michaels et al., 2024).

The study from Fuji et al. (2022) we also cited above. He focused on the Glucose transporter GLUT1 which plays a primary role in the glucose metabolism of cancer cells. He revealed that CTS such as Ouabain, Oleandrin, and Digoxin decreased the GLUT1 expression in the plasma cell membrane of diverse human cancer cells (liver cancer HepG2, colon cancer HT-29, gastric cancer MKN45 and oral cancer KB) hinting to an universal mechanism (which might be induced by EO) (Fujii et al., 2022).

Also linked to abnormal glycolysis in cancer cells is the Glycoprotein non-metastatic melanoma protein B (GPNMB), which is involved in invasion and metastasis.

Ono et al. (2016) revealed that high levels of GPNMB and Na^+^/K^+^-ATPase α1 isoform are associated with a poor prognosis in glioblastoma patients. They showed that GPNMB interacts with NKA α1 to activate PI3K/Akt and MEK/ERK pathways. However, it remains unclear whether the interaction of GPNMB and NKA α1 is involved in progression of glioma. The tumor size induced by the injection of glioma GL261 cells was larger in transgenic mice overexpressing GPNMB when compared with wild-type mice. Moreover, the interaction of GPNMB and NKA α1 was identified in the murine glioma model and in the tumors of glioblastoma patients. Ouabain suppressed the glioma growth induced by the injection of glioma cells in the transgenic mice overexpressing GPNMB and blocked the GPNMB-induced migration of glioma cells (Ono et al., 2016). These findings indicate that GPNMB promotes glioma growth via the NKA α1 isoform. Thus, the interaction between GPNMB and NKA α1 represents a novel therapeutic target for the treatment of brain glioblastomas.

The next author proceeds from the pivotal role of AMPK in the cell metabolism also from cancer cells. AMP-activated protein kinase (AMPK) is a central regulator of cellular energy homeostasis. The kinase is activated in response to stresses that deplete cellular ATP supplies such as low glucose, hypoxia, ischemia, and heat shock. Recently the ambiguous role of AMPK in cancer metabolism is under discussion.

Shen et al. (2020) showed before that Ouabain induces autophagic cell death in human lung cancer cells by regulating AMPK-mediated mTOR and Src-mediated ERK1/2 signaling pathways (Xu et al., 2009a). In the new study they revealed that treatment with Ouabain (25 nM) caused simultaneous activation of AMPK and Src signaling pathways in human lung cancer A549 and human breast cancer MCF7 cells (Shen et al., 2020). Co-treatment with AMPK siRNA greatly abrogates Ouabain-induced Src activation, whereas co-treatment with Src inhibitor PP2 has little effect on Ouabain-induced AMPK activity, suggesting that AMPK served as an upstream regulator of the Src signaling pathway. On the other hand, Ouabain treatment greatly depletes ATP production in A549 and MCF7 cells, and supplement of ATP (100 μM) blocked Ouabain-induced AMPK activation. Interestingly, Ouabain greatly inhibited the mitochondrial oxidative phosphorylation in the cancer cells, and exerted differential metabolic effects on glycolysis depending on cancer cell type. The last study we want to mention in this context is from Salyer et al. (2013) which we cited already above. In short, the authors concluded that cancer cells obviously exhibit fundamentally different metabolic pathways not only due to their high energy demand but in order to maintain their specific intracellular ion homeostasis (Salyer et al., 2013).

### 3.8 Effects of exogenous ouabain on drug resistance

Without doubt this review would not be complete without mentioning the issue of drug resistance induced by Ouabain and other CTS which indeed could contribute to the double-edge-sword effect in cancer therapy. We will cite here only two interesting studies, other you find below (Valente et al., 2007; Brouillard et al., 2001). Huang et al. (2002) reported for the first time that CTS (e.g., Ouabain and Digitoxin) induced resistance of human prostate cancer cells (PC-3) *in vitro* to tubulin-binding anticancer drugs, such as Paclitaxel, Vincristine and Vinblastine (Huang et al., 2002). Remarkably, CTS could reverse the G2/M arrest of the cell cycle and cell apoptosis induced by tubulin-binding agents. However, CTS showed little influence on the effects induced by Actinomycin D and doxorubicin, suggesting selectivity for microtubule-targeted anticancer drugs. Using *in situ* immunofluorescent detection of mitotic spindles, the authors showed that cardiac glycosides diminished paclitaxel-induced accumulation of microtubule spindles. Using an isotope-labeled assay method, they found that Ouabain inhibited the transport of [14C] Paclitaxel from the cytosol into the nucleus.

Riganti et al. (2009) revealed a crucial mechanism by which CTS induce P-glycoprotein (Pgp), a transmembrane transporter which extrudes several drugs, including anticancer agents like Doxorubicin (Riganti et al., 2009). In human colon cancer HT29 cells Ouabain and Digoxin increased the [Ca^++^](i) and this event was dependent on the calcium influx via the Na^+^/Ca^++^ exchanger. The increased [Ca^++^](i) enhanced the activity of the calmodulin kinase II enzyme, which in turn activated the transcription factor HIF-1α. The latter was responsible for the increased expression of Pgp. All the effects of glycosides were prevented by inhibiting the Na^+^/Ca^++^ exchanger or the calmodulin kinase II. The authors concluded that the efficacy of chemotherapeutic agent substrates of Pgp may be strongly reduced in patients taking Digoxin.

## 4 Discussion

The above data confirm with overwhelming unequivocal evidence the anti-tumor properties of Ouabain *in vitro* as well as *in vivo*. Out of 1.295 analyzed studies found by searching “Ouabain cancer” from 1970 to 2024 in Pubmed only three describe a pro-tumor effect *in vitro*, and mainly in leukemic cells. Interestingly, this effect was seen at very low (0–1 nM) Ouabain concentrations speaking in favor of our hypothesis that cancer cells according to their lower threshold (of sensitivity towards CTS) proliferate at very low Ouabain levels similar to normal cells at physiologic (higher) levels.

Besides, the data of the Ouabain effects on leukemic cells were contradictious. Whereas Wang et al. (2007) revealed that in 2 lymphocytic leukemia B95 and Jhhan cell lines low concentrations of Ouabain (<10 nmol/L) induced cell growth and upregulated the NKA α1 isoform at the plasma cell membrane, in 2 megakaryocytic leukemia M07e and Meg-01 cell lines, however, the same low dose of Oubain induced inhibition of cell growth and downregulation of the NKA α1 isoform (Wang et al., 2007). You could assume that the nature of these leukemic cell lines (lymphocytic vs. megakaryocytic) is responsible for this divergent reaction, but surprisingly, Xu et al. (2009b) observed that these low Ouabain concentrations (1 or 10 nmol/L) induced cell proliferation not only in the 2 lymphocytic (B95 and Jhhan) but also in the 2 megakaryocytic (M07e and Meg-01) leukemia cell lines (Xu et al., 2009b).

It is true that most if not all studies deal with the effect of exogenously applied Ouabain on cancer cell lines. We found only one study comparing the efficacy of Digoxin, Ouabain and mammalian-derived digoxin-like immune-reactive factor (DLIF) assumingly the endogenous OLC. Remarkably, Ihenetu et al. (2007) revealed that DLIF was about 10-fold more potent than (plant) Ouabain (IC50 1.9 nmol/L vs 26 nmol/L) at inducing apoptosis in Jurkat cells which hints to its important regulatory tasks at physiological serum concentrations in humans (50–1,000 pM) (Ihenetu et al., 2007).

Only one study in our screening over a span of more than 50 years, Yang et al. (2023), describes a negative interaction between endogenous Ouabain (EO) and the NKA α1 receptor through upregulation of the checkpoint ligand PD-L1 on lung cancer cells. Indeed, the study has solid data, *in vitro*, *in vivo* as well as from NSCLC patients and claims that by promoting tumor escape endogenous Ouabain is correlated with a bad prognosis. To summarize:

First, they saw in NSCLC patients an inverse relation between EO plasma levels and survival.

Second, they demonstrated that (exogenous!) Ouabain increases PD-L1 expression in mutant EGF-R lung cancer.

Third, they showed that NKA α1 overexpression at the plasma cell membrane is associated with decreased PD-L1 expression.

Fourth, they revealed in progressive lung cancer a reduced expression of NKA α1.

NKA α1 is co-localized with PD-L1 at the plasma cell membrane of lung cancer cells and they interact with each other via their cytoplasmatic domains. After activation of this NKA/PD-L1 complex PD-L1 is undergoing a clathrin-mediated endocytosis (CME) and lysosomal degradation. In NKA α1 silenced tumor cells the PD-L1- protein degradation was shown to be significantly delayed (Yang et al., 2023).

At this point, the authors see (themselves!) a dilemma–while the Ouabain effect on increased PD-L1 transcription is dependent on NKA α1 expression, the protein expression of NKA α1 was shown to decrease gradually with the duration of Ouabain treatment.

They solve this dilemma by postulating that EO deranges or inhibits the NKA α1/PD-L1 interaction.

They claim that sustained Ouabain treatment (or prolonged EO exposure? That is the question) downregulates NKA α1 to maintain (or: at the cost!) of up-regulating PD-L1 on the cell membrane of lung tumor cells.

More studies certainly are needed to verify these challenging data. Here we have the following comments:

The fact that high endogenous Ouabain levels are found in aggressive/progressive tumor patients does not mean automatically that Ouabain promotes tumor growth. There are abundant data about the anti-tumor properties of Ouabain. It rather could indicate that the individual in his trial to fight the cancer is up-regulating all endogenous Ouabain reserves to exorbitant levels, which actually have no physiologic meaning/activity anymore (shortly before “the fight is lost”).

In our own study with 84 breast cancer patients (poster presentation, Jerusalem 1999) we found a clear inverse correlation between EO/OLC plasma levels and lymph node metastasis, indicating a protective effect of endogenous Ouabain. The design of the study did not analyze the survival rate. But, remarkably, in a minority we found extremely upregulated OLC plasma levels (at least 10–30 fold above physiological ones) hinting to an excessive OLC production maybe as “a last struggle” (see above).

Another interesting (even if unlikely) scenario is a paraneoplastic production of EO especially in lung cancer or metastatic adrenal disease hence setting EO free as described by Goto et al. (1996).

Here certainly more studies dealing with the role of endogenous Ouabain in tumorigenesis and tumor progression are needed. But even so, it remains doubtful, why endogenous Ouabain which was proven identical to plant Ouabain in many including their (Yang et al.) own studies should have such an opposite (i.e., negative) effect on cancer as compared to exogenous Ouabain. The thesis of a harmful EO/NKA α1 interaction in PD-L1 + lung cancer would only make sense if EO and Ouabain are not identical (!). At this point, we want to mention again our planned project to develop an Ouabain aptamer which could help to analyze endogenous OLC levels more precisely and at the same time could track exogenous applied Ouabain.

Interestingly, Yang et al. (2023) found elevated EO levels only in immunological intact lung cancer models. After T-cell depletion the increase of EO was abolished, indicating that T cells are required for EO production in (lung) cancer. On the other hand, low EO levels seem to stimulate CD4 T-cell generation. This seemingly feed-back mechanism could be interpreted in a positive or negative way with respect to tumorigenesis. It corresponds to our findings in nude mice in which the basal adrenal EO content was reduced as compared to normal mice as well as plasma EO levels after application of stress stimuli (Weidemann et al., 2004). We concluded that the tumor growth in nude mice is facilitated by the dampened interaction between the immune (missing T-cells) and the endocrine (reduced EO) system.

Similarly, the fact that low expression of NKA α1 is seen by Yang et al. in aggressive progressive tumor patients can be interpreted in many different ways. We cited above several studies who described a different NKA isoform pattern in tumorous as compared to normal tissues with a change over time in the process of tumorigenesis.

For instance, it was revealed that in HCC the pattern of NKA isoforms changed as compared to non-malignant hepatic tissue with higher NKA α3 expression (Shibuya et al., 2010) indicating an active NKA α3 upregulation resp. NKA α1 downregulation process in cancer over time maybe due to endogenous Ouabain activity.

Altogether, the degree of expression of NKA α1 at the plasma cell membrane of cancer cells remains ambiguous and may dictate the fate towards tumor suppression as well as tumor progress.

On the one hand, as shown e.g., by Tian et al. (2009) the knockout of NKA α1 by siRNA is sufficient - not only in malignant but also benign cells - to induce cell growth inhibition (Tian et al., 2009). Similarly, Lefranc et al. (2008) claimed that targeting the NKA α1 isoform which is highly expressed in a majority of glioblastomas by novel CTS could represent a hallmark of anti-cancer therapy (Lefranc et al., 2008).

On the other hand, as shown by Banerjee et al. (2021) the loss of NKA α1 at the cell surface (of prostate cancer) induces epithelial-mesenchymal transition (EMT) and hereby facilitates resp. Promotes metastasis (Banerjee et al., 2021). Similarly, Salyer et al. (2013) found with increasing aggressivity of breast cancer cell lines e.g., in MDA-MB453 cells an absence of NKA α1, α3, and β1 on the protein as well as the mRNA level (Salyer et al., 2013).

We cited above the co-localization of NKA a1 and PD-L1 and the Clathrin-mediated endocytosis of PD-L1 (Yang et al., 2023). The authors also talk about the critical activation of the NKA-Src complex which causes not only transactivation of mutant EGF-R but is also involved in the CME of many components in the caveolae. Ouabain is an assumed activator of this NKA-Src complex - via binding to the NKA it releases the kinase domain of Src, increasing hereby the phosphorylation of Src at Y418, which trans-activates the EGF-R at Y845 and in turn activates the MAPK pathway (Tian et al., 2006). Liu and Shapiro (2007) analyzed in the renal LLC-PK1 cell line the role of the signalosome in the process of endocytosis and demonstrated that Ouabain-stimulated endocytosis of the NKA requires Caveolin-1 and Clathrin as well as the activation of c-Src, transactivation of EGF-R and activation of PI3K. They showed that c-Src, EGFR and the extracellular signal-regulated kinases 1 and 2 (ERK1/2) all were endocytosed along with the plasmalemmal NKA (Liu and Shapiro, 2007).

Hence, Ouabain could not only induce endocytosis of NKAα1 and EGF-R but also PD-L1. One decisive factor which decides about final endocytosis and degradation or repletion of survival factors at the plasma cell membrane could be the mutation status of EGF-R.

Yang et al. (2023) stressed that Ouabain increases PDL-1 only in lung cancer with mutant EGF-R. Ouabain seemingly induces different endocytotic trafficking and signaling pathways not only in benign vs. malignant cells but also in malignant cells according to the EGFR mutation status, the NKA isoforms and other not yet fully analyzed factors. While in benign cells a transactivation of wt-EGF-R via Src by Ouabain induces endocytosis of the signalosome and causes recycling of the NKA α1 subunit to the cell membrane via activation of the PI3-K/Akt/mTor pathway, in malignant cells a transactivation of EGF-R by Ouabain induces endocytosis of the signalosome and causes its lysosomal degradation, leading to NKA α1 depletion at the cell surface (Kometiani et al., 2005). This would explain the upregulation of PDL-1 but only when it is not undergoing endocytosis along with all other members of the caveolae.

Here we need to acknowledge that the data about Src/NKA/Ouabain interaction in cancer remain partly contradictious. As described above, some authors stress the importance of Src activation by Ouabain especially in the process of ROS and autophagy induction in lung cancer (Yan et al., 2015; Wang et al., 2015; Serrano-Rubi et al., 2020; Wang et al., 2009). Other authors claim that Src inhibition by Ouabain is mainly responsible for their anti-tumor activities (Xu et al., 2011; Chen et al., 2021; Hsu et al., 2015; Ono et al., 2016).

At this point, we will discuss a bit more the above mentioned seemingly contradictious effects of Ouabain on cell detachment/migration. The focal adhesion kinase (FAK) is involved in the Src-Akt/PI3K pathway. While Pongrakhananon et al. (2013) saw a under Ouabain treatment a downregulation of FAK, Akt and other migratory compounds (Pongrakhananon et al., 2013) Ruanghirun et al. (2014) saw a an upregulation of FAK with increased cell detachment (Ruanghirun et al., 2014). To mention, that increased cell detachment must not be considered *per se* only as negative, as it may induce apoptosis/anakoisis, but in general, it facilitates metastasis. In both studies, they used human lung cancer cell lines (H292 and H23) but slightly different dosages of Ouabain (10–30 pmol/L vs. 0–10 pmol/L). These minimal differences in dosage might be not relevant for the opposite effect of Ouabain but highlights the importance of choosing the appropriate dosage according to the known dual activity inherent in Ouabain, in benign as well as malign cells.

It is important to mention that recent studies doubt the direct interaction of Src/NKA/Ouabain. Gable et al. (2014) reexamined the suggested interaction between Src and Na^+^/K^+^-ATPase α1 isoform. They revealed that under physiological conditions Src is not in contact with NKA α1 and that Src obviously is unnecessary for Ouabain-induced cell signaling (Gable et al., 2014). To a similar conclusion (that at least not all NKA isoforms form a complex with Src) came another group. Madan et al. (2017) observed that the NKA α3 isoform does not interact with Src and EGF-R, but rather, that the interaction between Ouabain and NKAα3 activates PI3-K directly with down-stream increasing p-Akt and ERK1/2 levels (Madan et al., 2017). Similarly, Wu et al. (2013) concluded that the PI3-K activation by Ouabain is independent of Src and EGF-R assuming an Ouabain-induced direct interaction of a proline-rich domain of the α3-subunit of the NKA with the SH3 domain of the p85 subunit of PI3-K1A (Wu et al., 2013). In summary, here further research will be necessary, to find the precise interactions between the different NKA isoforms and their cytosolic neighboring compounds. It is even possible that opposite actions are triggered by different domains of the NKAα1 isoform. The group of Tian/Li/Xie demonstrated that upon Ouabain binding a transformational change of the NKAα from E1 to E2 is induced leading to the release of the N domain of NKAα1 from the Src kinase which hereby is activating Src while the second cytosolic domain (CD2) of NKAα1 inhibits Src directly by binding to SH2 (Banerjee et al., 2015).

The interaction between Ouabain and the immune system is fascinating but not yet sufficiently analyzed. Whereas Yang et al. (2023) claim that EO/NKA α1 interaction increases tumor escape we found many studies dealing with a favorite impact of EO/Ouabain on the immune system. We mentioned above the work of Shandell et al. (2022) who saw a downregulation of the immune checkpoint protein IDO1 at mRNA and protein levels mediated by Ouabain via NKA inhibition (Shandell et al., 2022). The studies we cited above found an Ouabain-induced reduction especially of Tregs. Tregs are known to exert immunosuppressive effects and tumorigenesis is often accompanied by an increase in Treg cells. Interestingly, in recent years Ouabain and other CTS gain interest also as Co-factors to induce a robust immunogenic cell death. As Xiang et al., 2020 demonstrated a combination of Cisplatin and Digoxin not only led to complete immunogenic cell death of tumor cells but these cells also could serve as vaccines against the same tumor (Xiang et al., 2020).

Last but not least, the interplay between Ouabain and cancer metabolism remains controversial and elusive. Whereas many studies (from the 70ies) provided solid data that Ouabain is down-regulating cancer metabolism mainly via inhibition of glucose uptake and glycolysis the partly activating effects of Ouabain on some signaling pathways could indeed promote tumor growth. We mentioned the work of Wang et al. (2015) who revealed an activation of AMPK-Src pathways by Ouabain in the process of inducing autophagy in lung cancer cells (Wang et al., 2015). But AMPK is known to stimulate pathways (e.g.,) glucose uptake which are critical for cell survival during metabolic stress like e.g., hypoxia in tumor microenvironment. On the other side, AMPK can protect against cell damage due to oxidative stress, which in turn protects against tumor initiation. In summary, in cancer, the role of AMPK exhibits two opposite faces just like a double-edged sword. This might be the dilemma of many molecules involved in the signaling pathways we discussed here, including Ouabain resp. EO.

## 5 Hypothesis

In view of all these partly contradicting data we want to present a hypothesis.

1. At the surface of the plasma cell membrane tumor cells primarily express the survival promoting NKA α1 isoform. It co-localizes with Src, PDL-1, EGF-R and other growth-stimulating compounds.
2. Endogenous Ouabain is down-regulating NKA α1 by endocytosis and lysosomal degradation and instead is up-regulating the more EO-sensitive NKA α3 isoform at the plasma cell membrane for better tumor defense.
3. But NKA α3 expression could also result in increased Src-ERK1/2 and PI3K/Akt activation which may be harmful for the individual. Only a sustained ERK1/2 activation seems to induce cell growth arrest.
4. Hence, also the NKA α3 might undergo endocytosis upon Ouabain binding reflected in the increased amount of intracellular vesicles expressing the NKA α3 especially in tumor cells.
5. Thus, in the evolution of tumorigenesis the expression of NKA α3 was split to exert its anti-tumor effects at the cell surface as well as intracellular.
6. But tumor cells who miss the upregulation of NKA α3 (especially in lung carcinoma with high PDL-1 expression) EO/Ouabain could further increase the expression of PDL-1.
7. In summary, it seems that a fine tuned balance between the expression of NKA isoforms (and other receptors) at the plasma cell membrane–which might be modulated by EO–is crucial for the fate of tumor cells, to enter the survival or the death pathway.

**FIGURE 1:**
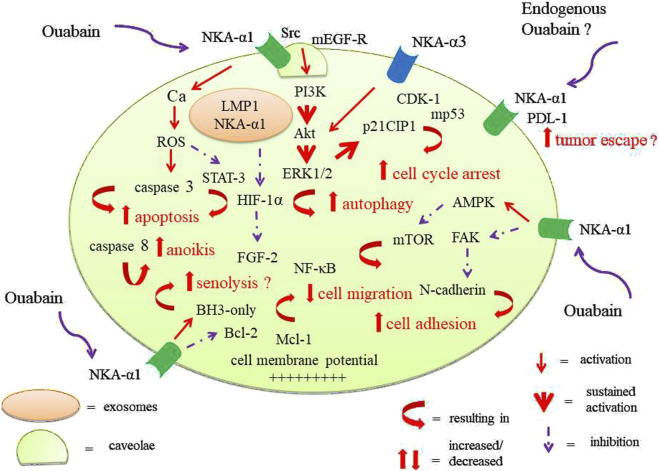
Different effects of Ouabain on tumor cells. Ouabain modulates mainly via the NKA-α1/Src/EGF-R complex in caveolae many signaling pathways and induces dose- and time dependent decreased tumor cell survival, cell migration and adhesion but rarely may result in e.g., tumor escape. Ouabain can circumvent the Src/EGF-R complex via direct interaction NKA-α3 and PI3/Akt. The pathway of senolysis seems to involve pro-apoptotic proteins.

The multiple complex activities of Ouabain/EO are summarized in Figure 1.

## 6 Conclusion

We want to conclude with the following statement assuming that EO are identical with Ouabain. While the anti-tumor effects of Ouabain are consistently shown in many, nearly all tumor entities, *in vitro* as well as *in vivo*, it seems that the results in non-small cell lung cancer must be considered with caution.

Even there are robust data showing that Ouabain can induce especially in lung cancer autophagy (via the activation of the Src-ERK1/2 pathway), there is obviously a high risk of inducing via the same signaling pathways, maybe even in parallel, survival pathways e.g., increased cell detachment, EMT or AMPK-activation and last, but not least, PD-L1 upregulation.

Thus, as long as these contradictious pathways in NSCLC are not completely revealed, we would recommend advancing and promoting the application of Ouabain in all the other mentioned tumor entities especially Breast cancer and Glioblastoma.
